# NMR Characterization of the Interaction of the Endonuclease Domain of MutL with Divalent Metal Ions and ATP

**DOI:** 10.1371/journal.pone.0098554

**Published:** 2014-06-05

**Authors:** Ryota Mizushima, Ju Yaen Kim, Isao Suetake, Hiroaki Tanaka, Tomoyo Takai, Narutoshi Kamiya, Yu Takano, Yuichi Mishima, Shoji Tajima, Yuji Goto, Kenji Fukui, Young-Ho Lee

**Affiliations:** 1 Institute for Protein Research, Osaka University, Suita, Osaka, Japan; 2 RIKEN SPring-8 Center, Harima Institute, Sayo-cho, Sayo-gun, Hyogo, Japan; Wake Forest University, United States of America

## Abstract

MutL is a multi-domain protein comprising an N-terminal ATPase domain (NTD) and C-terminal dimerization domain (CTD), connected with flexible linker regions, that plays a key role in DNA mismatch repair. To expand understanding of the regulation mechanism underlying MutL endonuclease activity, our NMR-based study investigated interactions between the CTD of MutL, derived from the hyperthermophilic bacterium *Aquifex aeolicus* (aqMutL-CTD), and putative binding molecules. Chemical shift perturbation analysis with the model structure of aqMutL-CTD and circular dichroism results revealed that tight Zn^2+^ binding increased thermal stability without changing secondary structures to function at high temperatures. Peak intensity analysis exploiting the paramagnetic relaxation enhancement effect indicated the binding site for Mn^2+^, which shared binding sites for Zn^2+^. The coexistence of these two metal ions appears to be important for the function of MutL. Chemical shift perturbation analysis revealed a novel ATP binding site in aqMutL-CTD. A docking simulation incorporating the chemical shift perturbation data provided a putative scheme for the intermolecular interactions between aqMutL-CTD and ATP. We proposed a simple and understandable mechanical model for the regulation of MutL endonuclease activity in MMR based on the relative concentrations of ATP and CTD through ATP binding-regulated interdomain interactions between CTD and NTD.

## Introduction

DNA mismatch repair (MMR) is the process by which post-replicative base pair errors are rectified, thereby enhancing the fidelity of DNA replication 100–1000 fold [1]. Mutations in MMR genes have been shown to cause hereditary non-polyposis colorectal cancer, referred to as Lynch Syndrome [2], [3]. Although significant differences have been reported between *Escherichia coli (E. coli)* and eukaryotic MMR, the basic concepts of MMR have been developed primarily through research on *E. coli* [4], [5]. MMR starts from the recognition of mismatched base pairs by MutS, and is followed by the recruitment of MutL by the mismatch:MutS complex.

The mismatch:MutS:MutL complex has been shown to initiate downstream repair machinery in both *E. coli* and eukaryotes, in which newly synthesized DNA strands are specifically nicked at either side of the mismatch [6], [7]. However, the mechanisms underlying daughter strand recognition and incision are decisively different between *E. coli* and eukaryotes. While *E. coli* MMR possesses MutH endonuclease, which cleaves daughter strands at hemi-methylated GATC sites, eukaryotes and most bacteria lack MutH; hence, the counterpart of MutH in eukaryotic and eubacteria species was a topic for speculation until the latent endonuclease activities of human and yeast MutLα were demonstrated in the presence of divalent metal ions such as Mn^2+^ [8], [9].

Since MutL lacks base specificity as an endonuclease, understanding the regulation mechanism of MutL endonuclease activity is one of the central themes that has been focused on to expand knowledge on MMR. MutL consists of a C-terminal dimerization domain (CTD) and N-terminal ATPase domain (NTD) connected with disordered linker regions [10], [11]. MutL has been shown to form heterodimers and homodimers in eukaryotes and prokaryotes, respectively [5].

Alterations in the quaternary structure of MutL were assumed to result from interdomain interactions between the NTD and CTD/NTD, and the NTD may be involved in regulating endonuclease activity when it binds with metal ions or ATP [11]. Isolated aqMutL-NTD was previously shown to stimulate the endonuclease activity of aqMutL-CTD in a zinc-dependent manner [12]. A recent study also indicated that MutS coupled with the endonuclease activity of MutL stimulated by the DNA mismatch in an ATP-dependent manner, and also that the NTD of MutL primarily mediated the interaction between MutL and MutS. Therefore, MutS may stimulate the endonuclease activity of MutL through interdomain interactions between the CTD and NTD [13]. However, the functional details associated with changes in the quaternary structure remain largely unknown [11], [12], [14], [15].

Therefore, the intermolecular interactions between MutL and various binding molecules such as divalent metal ions, ATP, other MMR proteins, and DNA based on MutL conformations need to be determined in order to examine the regulation of MutL endonuclease activity. Recent structural studies using X-ray crystallography have provided information on CTD conformations in *Bacillus subtilis* MutL (bsMutL). The static crystal structures of the CTD:Zn^2+^ complex from *Neisseria gonorrhoeae* and the complex of CTD from *Saccharomyces cerevisiae*, with fragments containing the MIP-box motifs of the Exo1 and Ntg2 proteins [1], [16], [17], revealed the detailed interacting sites and binding modes of the CTD for partner molecules.

However, the moderate and/or weak intermolecular interactions and conformational dynamics of MutL, which are key to understanding MMR in solution, have been difficult to detect by X-ray crystallography. Therefore, solution-state NMR is a powerful approach used to examine the intermolecular interactions between MutL and other molecules because NMR provides residue-based information, even on weak intermolecular interactions and protein dynamics in solution.

We here described an NMR investigation of the CTD of aqMutL-CTD, and its intermolecular interactions with molecules such as Zn^2+^, Mn^2+^, ATP, and DNA, which have been suggested to interact with the CTD. Based on the almost complete NMR backbone assignment of aqMutL-CTD (∼97% of 103 assignable residues), the model structure of aqMutL-CTD, molecular docking simulations, and NMR titration/relaxation measurements, we revealed the intermolecular interaction and binding sites of aqMutL-CTD for binding partners. We further addressed the biological implications of Zn^2+^ and Mn^2+^ binding and a possible mechanical model for the regulation of MutL endonuclease activity.

## Materials and Methods

### Protein Expression and Purification

aqMutL-CTD was expressed and purified as previously described [12]. *E. coli* was cultured in minimal media containing ^13^C-glucose and ^15^N-NH_4_Cl as the sole carbon and nitrogen sources, respectively. The cell body was collected by centrifugation and lysed by sonication in 50 mM Tris-HCl (pH 7.8) containing 500 mM NaCl. After centrifugation at 18,000 rpm for 60 min, the supernatant was heated at 70 °C for 10 min. The heated solution was again centrifuged and the supernatant was loaded to the DEAE column (TOSOH, Tokyo, Japan). The pass solution was then loaded onto the SP-sepharose column (GE-Healthcare Biosciences, USA) pre-equilibrated with 50 mM Tris-HCl buffer (pH 7.8) containing 1 mM dithiothreitol (DTT). Ammonium sulfate was added to the collected fraction to yield a final concentration of 1M. The solution was again loaded onto the Toyopearl-phenyl column (TOSOH, Tokyo, Japan). The fractions of aqMutL-CTD were collected and concentrated using an Amicon Ultra centrifugal filter (Millipore, Billerica, USA). The sample solution was then applied to the superdex 75 HR column (GE Healthcare Biosciences, USA) pre-equilibrated with 50 mM potassium phosphate buffer (pH 6.8) containing 100 mM KCl, 1 mM DTT, and 1 mM EDTA. The fractions of aqMutL were collected and their purities were checked by SDS-PAGE (>95%). Protein concentrations were determined by the molar extinction coefficients of aqMutL, 19005 M^−1^cm^−1^ at 278 nm [18].

### Sequential Backbone Assignment of aqMutL-CTD

aqMutL-CTD samples uniformly labeled with ^2^H-^13^C-^15^N, ^13^C-^15^N, or ^15^N were prepared using 50 mM potassium phosphate buffer (pH 6.8) containing 100 mM KCl, 5 mM DTT, 1 mM EDTA, 10% D_2_O, and 0.02% NaN_3_. Potassium phosphate buffer was used to assign aqMutL-CTD because the dispersion and sharpness of the spectra obtained from samples in potassium phosphate buffer were better than those in Tris-HCl buffer (Figure S1). All NMR measurements for backbone assignments were performed at 40°C in an AVANCE II-800 spectrometer equipped with a cryogenic probe (Bruker BioSpin, Germany). Data were processed by NMR-Pipe [19] and analyzed by Sparky. Sequential backbone assignments of aqMutL-CTD were performed primarily on uniformly ^15^N-^13^C-labeled aqMutL-CTD using a set of three-dimensional (3D) heteronuclear coherence transfer measurements of HNCO, HNCACO, CBCACONH, and HNCACB. Histidine-specific isotope labeling was introduced because several unassigned peaks, especially around H400 and H404 in the α2-β4 loop region, hampered the complete sequential backbone assignment (Figure S2). This method allowed us to assign all three histidine residues: H353, H400, and H404. The remaining unassigned peaks could be sequentially assigned based on the assignment information of histidine residues and TROSY-HNCACB and TROSY-CBCACONH measurements of ^2^H-^15^N-^13^C-labeled samples.

**Figure 1:**
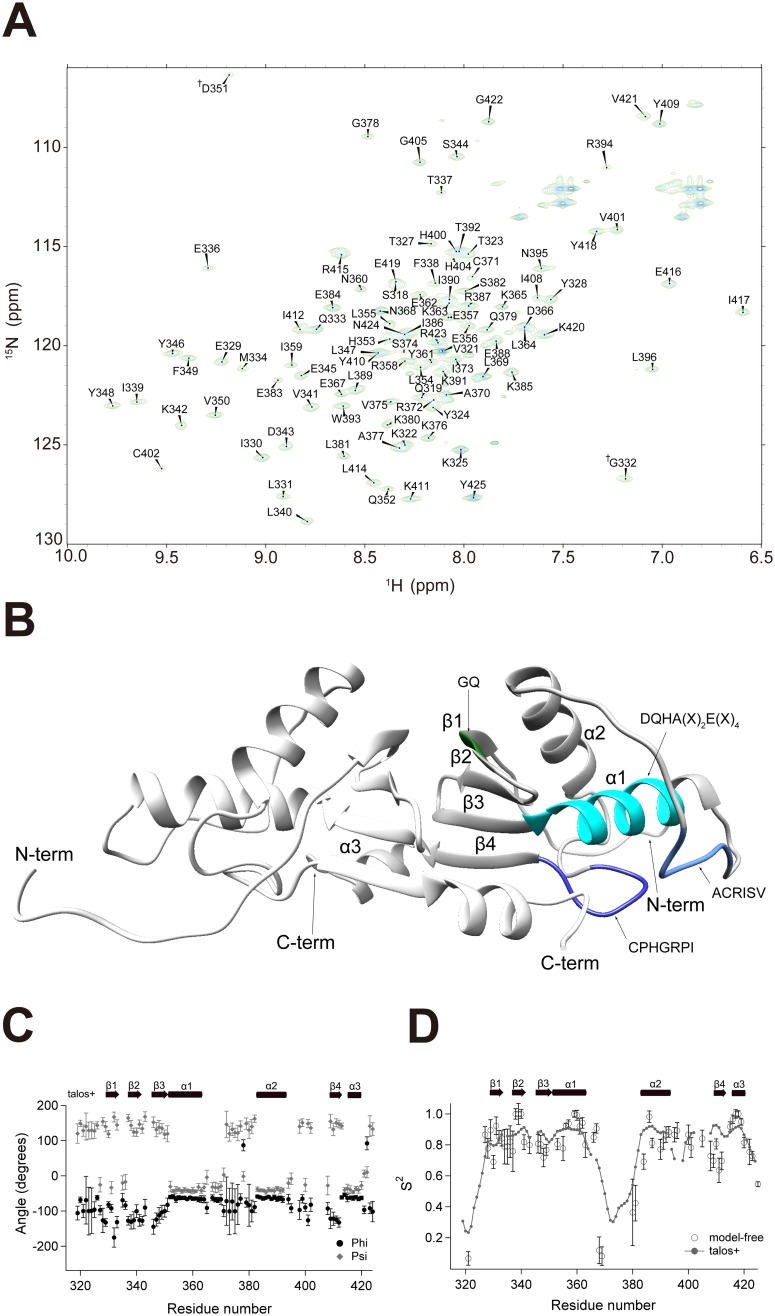
The two-dimensional NMR spectrum and model structure of aqMutL-CTD. (A) The results of the sequential backbone assignment of aqMutL-CTD (aqMutL_316–425_) are displayed in the ^1^H-^15^N heteronuclear single quantum coherence (HSQC) spectrum. Cross-peaks are labeled using the one-letter amino acid code and residue number. The aliased peaks (G332 and D351) on the ^15^N axis are indicated with ″†″. (B) The model structure of the homodimer of aqMutL-CTD was generated by Modeller (see the Materials and Methods). The α-helix and β-strand and both termini are labelled. (C) The phi (sphere) and psi (diamond) angles and order parameters (*S* ^2^), on the left and right axes, respectively, are plotted against the residue number. The α-helix (arrow) and β-strand (rectangle) predicted using TALOS+ (open) and Modeller (closed) are shown above. (D) The values of *S* ^2^ obtained from the model-free analysis (open circle) and from TALOS+ (solid circle plus line) are plotted against the residue numbers.

Assignment results were confirmed by the ^1^H-^15^N HSQC spectra of inversely- and arginine/lysine specifically-labeled samples (Figure S3). The final assignment rate was ∼97%, excluding the 7 proline residues and N-terminal residue. Unassigned residues were L317, D335, L397, N398, and R406, which were located in the unstructured loop regions of the model structure of aqMutL-CTD (Figure 1A). The assignment result was depicted on the ^1^H-^15^N HSQC spectrum of aqMutL-CTD (Figure 1A) and final resonance assignments were deposited to BioMagRes DB (BMRB accession number 11545).

### NMR Relaxation Measurement of aqMutL-CTD and Model-free Analysis

^15^N longitudinal and transverse relaxation times (*T*_1_ and *T*_2_) and ^1^H-^15^N steady state NOE values were measured using a 0.4 mM aqMutL-CTD sample in the same buffer used in the backbone assignment of aqMutL-CTD using a Bruker DRX-500 spectrometer equipped with shielded triple-axis gradient triple-resonance probes at 40°C (Figure S4). *T*_1_ was measured with relaxation delays of 5, 150, 300, 450, 600, 800, 1000, 1200, 1400, 1600, and 1800 ms. *T*_2_ was measured with relaxation delays of 7.2, 21.6, 43.2, 64.8, 86.4, 108.0, 129.6, 151.2, 180.0, 208.8, and 237.0 ms. Single exponential curve fitting was performed using Sparky to obtain the values of *T*_1_ and *T*_2_. ^1^H-^15^N NOE values were calculated by comparing the peak intensities with and without ^1^H saturation of 3 s. The global tumbling time (τ_m_) of aqMutL-CTD was estimated to be 11.7 ns using the *T*_1_/*T*_2_ values. By incorporating the three relaxation parameters (*T*_1_, *T*_2_, and NOE) and τ_m_ value to the model-free analysis with Tensor 2 [20], the values of the order parameters (*S* ^2^) were obtained under the assumption of isotropic tumbling of the CTD.

### NMR Titrations of aqMutL-CTD with Various Molecules

Changes in the NMR signals of the ^1^H-^15^N HSQC spectra of uniformly ^15^N-labled CTD with the addition of other molecules (Zn^2+^, Mn^2+^, ATP, and ADP) were monitored using the AVANCE II-800 or AVANCE III-950 spectrometer (Bruker BioSpin, Germany) equipped with a cryogenic probe at 40°C. Measurements were performed using 0.1 mM CTD in 50 mM Tris-HCl or potassium phosphate buffer (pH 7.0) containing 100 mM KCl, 1 mM DTT, 0.02% NaN_3_, and 10% D_2_O.

The titration of Zn^2+^ was performed using the 25 mM ZnCl_2_ stock solution. Aliquots of the stock solution were added to the CTD solution to reach the ratios of [ZnCl_2_]/[CTD] = 0, 0.4, 0.8, 2.0, and 2.4. Similarly, 50 mM MnCl_2_ of the stock solution was prepared, and aliquots of the stock solution were added to aqMutL-CTD in phosphate buffer to reach the ratios of [MnCl_2_]/[aqMutL-CTD] = 0, 0.2, 0.4, 0.8, 2.0, and 4.0. The peak intensity ratio was calculated using the intensity of the peaks without MnCl_2_ as a reference. The stock solution of 100 mM ATP and ADP was prepared, and aliquots of the stock solution were added to the CTD solution to reach the ratios of [ATP]/[CTD] or [ADP]/[CTD] = 0, 20, 40, 60, 80, and 100. The excess addition of ATP over the ratio of 120 resulted in the precipitation of the protein sample. Synthesized 21-mer singled-stranded DNA, 5′-GCGGTCATAGTCAAGATACCG-3′ (Integrated DNA Technologies, Inc.) was annealed to complementary single-stranded DNA (Integrated DNA Technologies, Inc.) to obtain 21-bp double-stranded DNA (dsDNA) and the stock solution of 6 mM 21-bp dsDNA was prepared as previously described [21]. Aliquots of the stock solution of dsDNA were added to 0.1 mM CTD in 25 mM Tris-HCl buffer containing 25 mM KCl, 1 mM EDTA, 1 mM DTT, 0.02% NaN_3_, and 10% D_2_O to reach the ratios of [dsDNA]/[CTD] = 0, 4, 8, and 12.

Chemical shift differences in the cross-peaks by titration were calculated using the relationship:
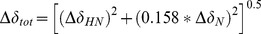
where Δ*δ*_HN_ and Δ*δ*_N_ are changes in the ^1^H and ^15^N chemical shifts in ppm, respectively. The weighting factor of 0.158 was used to adjust the relative magnitudes of the amide nitrogen chemical shift range and the amide proton chemical shift range.

### Model Structure of aqMutL-CTD and Its Complexes with Zn^2+^ and Mn^2+^

The model structure of aqMutL-CTD was built using the homology modeling methodology by Modeller 9.12 [22]. The X-ray crystal structures of bsMutL (PDB ID: 3KDG) and its complex with Zn^2+^ (PDB ID: 3KDK) were used as a template structure, with the secondary structure restraints obtained from the TALOS+ secondary structure prediction based on NMR chemical shift assignment values.

We further generated the model structures of aqMutL using bacterial NgoL MutL-CTD homodimer (PDB ID: 3NCV) and eukaryotic PMS1-CTD in the MutLα-CTD heterodimer (PDB-ID: 4E4W) for a template X-ray structure to assess the reliability of the homology modeling (Figure S10). Then, we compared these two model structures with the model structure generated using bsMutL-CTD. The overall conformation of the model structure with the bacterial X-ray structure is quite similar to that with bsMutL-CTD except for a few residues in the α-helix (α2 in the model structure based on bsMutL-CTD) and the β-strand (β3 in the model structure based on bsMutL-CTD). The slight structural differences in the loop structures were also found. On the one hand, the other model structure generated with the eukaryotic X-ray structure critically differs with the lack of the α-helix in the C-terminus of CTD (α4 in the model structure based on bsMutL-CTD) and the additional α-helical component in the N-terminus of CTD. The model structure of aqMutL-CTD generated using bsMutL-CTD was selected for this study due to the highest identity of the primary sequence (∼32%) between aqMutL-CTD and bsMutL-CTD.

A graphical representation of the putative Mn^2+^ binding site of aqMutL-CTD, based on the NMR peak intensity analysis, was depicted by referencing the previously published X-ray crystal structure of Mn^2+^-bound protein (PDB ID: 1N8F), which was shown to possess a similar coordination geometry for Mn^2+^ binding using the 3D-motif search capability of Swiss Pdb-Viewer [23].

### Docking Simulation of the Complex of CTD with ATP

A docking simulation was performed using myPresto/Sievgene based on the results of the NMR chemical shift perturbation analysis of ATP binding to aqMutL-CTD [24]. The ligand molecule was prepared in Sybyl mol2 file format. The atomic charges of ATP were obtained from the following web site: [http://www.pharmacy.manchester.ac.uk/bryce/amber/]. The force field parameters for the protein were similar to those in AMBER parm99 [25].

### Circular Dichroism (CD) Spectroscopy

Far-UV CD spectra of 20 µM (0.27 mg ml^−1^) aqMutL-CTD in 50 mM Tris-HCl (pH 7.0) including 100 mM KCl were recorded in the absence and presence of 40 µM Zn^2+^, 80 µM Mn^2+^, and 2 mM ATP at 40°C, respectively. Heat scanning of aqMutL-CTD from 40 to 95–100°C was also performed in the absence and presence of divalent metal ions and ATP under the same concentration settings described above by monitoring CD signals at 220 nm at a rate of 1°C min^−1^. CD measurements were performed with a J-720 spectropolarimeter (Jasco, Japan) using a cell with a light path of 1 mm. CD signals between 195 and 250 nm were expressed as the mean residue ellipticity [θ] (deg cm^2^ dmol^−1^). Temperature was regulated using a PTC-423L Peltier-unit (Jasco, Japan).

### Isothermal Titration Calorimetry (ITC) Measurement

CTD solution was dialyzed against 50 mM Tris-HCl (pH 7.0) containing 100 mM KCl and 1 mM β-mercaptoethanol which used instead of DTT for minimizing baseline drift of thermogram. ATD and ADP were dissolved by using the buffer after dialysis and their concentrations were determined by UV-vis absorbance. All of the samples were degassed for 3 min at 40°C before being loaded into the calorimeter. Calorimetric experiments were performed with a VP-ITC instrument (GE-Healthcare Biosciences, USA) at 40°C. The nucleotide (2 mM ATP or ADP) in the injection syringe was titrated into 16 µM CTD in the ITC cell (Figure S8). Titration experiments consisted of 39 injections spaced at intervals of 400 sec. The injection volume was 7 µl and the cell was continuously stirred at 242 rpm.

### Light Scattering Measurements

Light scattering of all of the sample solutions was measured at 40°C using a Hitachi fluorescence spectrophotometer F4500 (Hitachi, Tokyo, Japan) with the excitation wavelength at 350 nm (Figure S9). CTD and ATP were dissolved in 50 mM Tris-HCl (pH 7.0) containing 100 mM KCl and 1 mM β-mercaptoethanol. The amyloid fibrils of Aβ_1–40_ peptides were also prepared for the controls of light scattering of aggregates. Aβ_1–40_ peptides were dissolved and used for the formation of amyloid fibrils as described in the previous study [26]. The formation of Aβ_1–40_ fibrils was confirmed by the far-UV CD spectroscopy and thioflavin T assay (data not shown).

## Results

### Characterization of aqMutL-CTD Stereostructures

**Figure 2:**
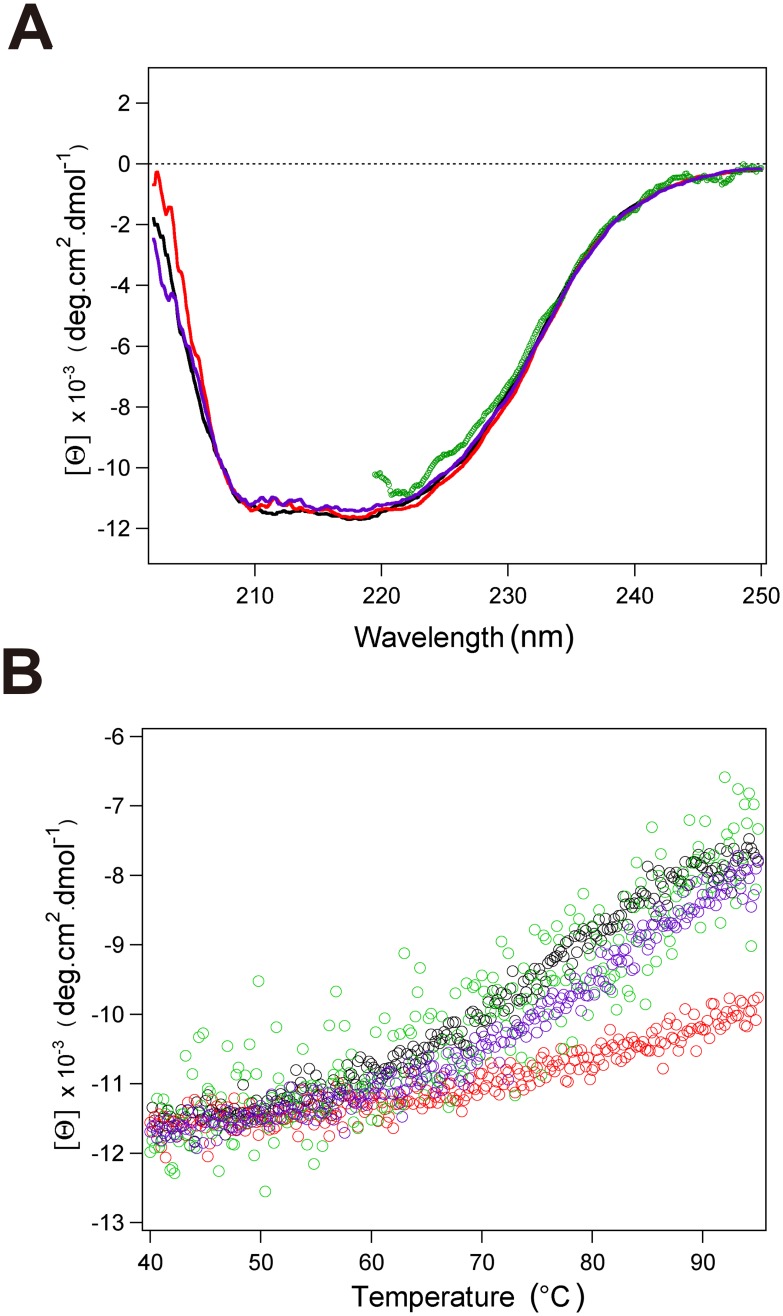
CD measurements of aqMutL-CTD in the presence and absence of binding partners. (A) The far-UV CD spectra of aqMutL-CTD without (black) and with Zn^2+^ (red), Mn^2+^ (purple), and ATP (green) are shown. Noisy data points in the shorter wavelength from 220 nm in the presence of ATP were omitted. (B) Heat scans of aqMutL-CTD are shown without (black) and with Zn^2+^ (red), Mn^2+^ (blue), and ATP (green) using the CD signal at 220 nm.

The ^1^H-^15^N HSQC spectrum displayed well-dispersed cross peaks, which indicated that a homodimer of aqMutL-CTD is a solid 3D structure at pH 6.8 and 40°C (Figure 1A). The far-UV CD spectrum of aqMutL-CTD showed a minimum from ∼210 nm to ∼220 nm, indicating structured secondary structures (Figure 2A). A homology model of an aqMutL-CTD conformation was produced using Modeller 9.12 to obtain detailed information on the tertiary structure (Figure 1B) [22]. bsMutL-CTD (PDB ID: 3KDG) [1] was used as the template conformation for modeling because bsMutL-CTD showed ∼32% sequence identity to aqMutL-CTD, which was sufficient for reliable homology modeling [27]. The amino acid sequence and conserved motifs of various MutL from different species are shown (Figure S5). The homology model was generated under the constraints of the secondary structures predicted by TALOS+ [28], estimating dihedral angles based on the assigned chemical shift information of C_O_, Cα, C_β_, HN, and ^15^N of aqMutL-CTD (Figure 1C). The produced model of aqMutL-CTD formed an inverted homodimer with an interface consisting of four β-sheets (β1–β4), each abundantly incorporating hydrophobic residues, and one α-helix (α3), domain swapping with each other in the aqMutL-CTD dimer (Figure 1B). Helix α1, the α1–α2 loop, and the α2–β4 loop included the conserved motifs DQHA(X)_2_E(X)_4_, ACRISV, and CPHGRPI, respectively.

The *S* ^2^ value ranged from 0 to 1 depending on the degree of flexibility and higher *S* ^2^ values reflected lower flexibility in the fast time scale. Thus, *S* ^2^ could be a reporter for protein dynamics and structure. High *S* ^2^ values (over 0.7) in the α-helix and β-strand and low *S* ^2^ values (below 0.7) in unstructured regions of the loops and terminal parts were consistent with the predicted secondary structural elements (Figure 1D). The loop between the α1 and α2 helices showed high *S* ^2^ values of approximately 0.8, which indicated a rigid backbone conformation.

### Zn^2+^-Bound aqMutL-CTD Displayed Enhanced Thermostability

Zn^2+^ was previously suggested to be involved in the regulation of MutL endonuclease activity for proper MMR *in vitro* and *in vivo* [1], [8], [9] and is related to the interdomain interactions between the CTD and NTD [12], [15].

**Figure 3:**
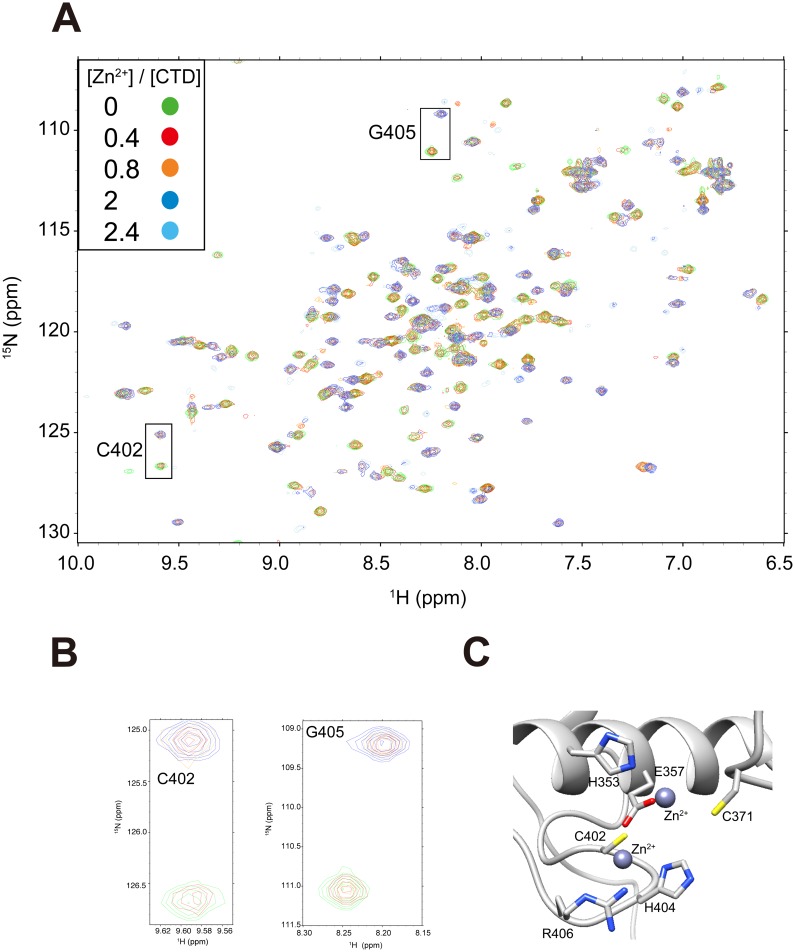
The characterization of interactions between aqMutL-CTD and Zn^2+^. (A) The ^1^H-^15^N HSQC spectra of 100 µM aqMutL-CTD at the various concentrations of Zn^2+^ are superimposed. The representative peaks (C402 and G405) showing the slow exchange are indicated with the one-letter amino acid code and residue number. (B) The change in the chemical shift of C402 and G405. (C) Putative binding sites of Zn^2+^ are magnified (see the Materials and Methods). The side chains of H353, E357, C371, C402, and H404 are shown. The blue spheres indicate Zn^2+^ ions.

The NMR-based titration of Zn^2+^ was performed to examine the intermolecular interactions between Zn^2+^ and aqMutL-CTD in solution. We observed large changes in the peaks of the ^1^H-^15^N HSQC spectrum: many new peaks appeared primarily because of slow exchanges and simultaneously broadened due to intermediate exchanges; however, we could not assign all new peaks. Representative residues in the slow exchange regime, C402 and G405, are indicated by rectangles (Figures 3A and 3B). These results suggested that Zn^2+^ binding was tight and related to the relatively broad regions of aqMutL-CTD, which may be involved in a conformational change. The putative Zn^2+^ binding sites of aqMutL-CTD indicated by the reported X-ray crystallography of bsMutL-CTD were also illustrated (Figure 3C). The modeled complex structure of the CTD and Zn^2+^ provided putative residues that interacted with the two Zn^2+^ ions: H353 and E357 in DQHA(X)_2_E(X)_4_ of the α1 helix, C371 in ACRISV, and C402, H404, and R406 in the CPHGRPI motif.

The far-UV CD spectrum of aqMutL-CTD was obtained in the presence of Zn^2+^ to further characterize the Zn^2+^-bound state of aqMutL-CTD. No change was observed in the CD spectrum, which revealed that Zn^2+^ binding did not perturb the secondary structures (Figure 2A). The thermal denaturation of CTD in the presence and absence of Zn^2+^ was examined by tracing CD signals at 220 nm (Figure 2B). The results of thermal denaturation experiments demonstrated that Zn^2+^ binding significantly enhanced the thermostability of aqMutL-CTD. Free CTD began to melt cooperatively at ∼50°C and was almost completely denatured at ∼95°C. Meanwhile, Zn^2+^-bound CTD largely changed the thermal denaturation profile. CTD melted gradually from ∼65°C, and heat denaturation was not finished even at ∼95°C.

### Determination of the Mn^2+^ Binding Sites of aqMutL-CTD in Solution

Recent endonuclease assays have suggested that Mn^2+^ is essential to activate the endonuclease activity of aqMutL-CTD *in vitro* [12]. Other studies have also clarified that human and yeast MutLα share a dependence on Mn^2+^ endonuclease activity [8], [9].

**Figure 4:**
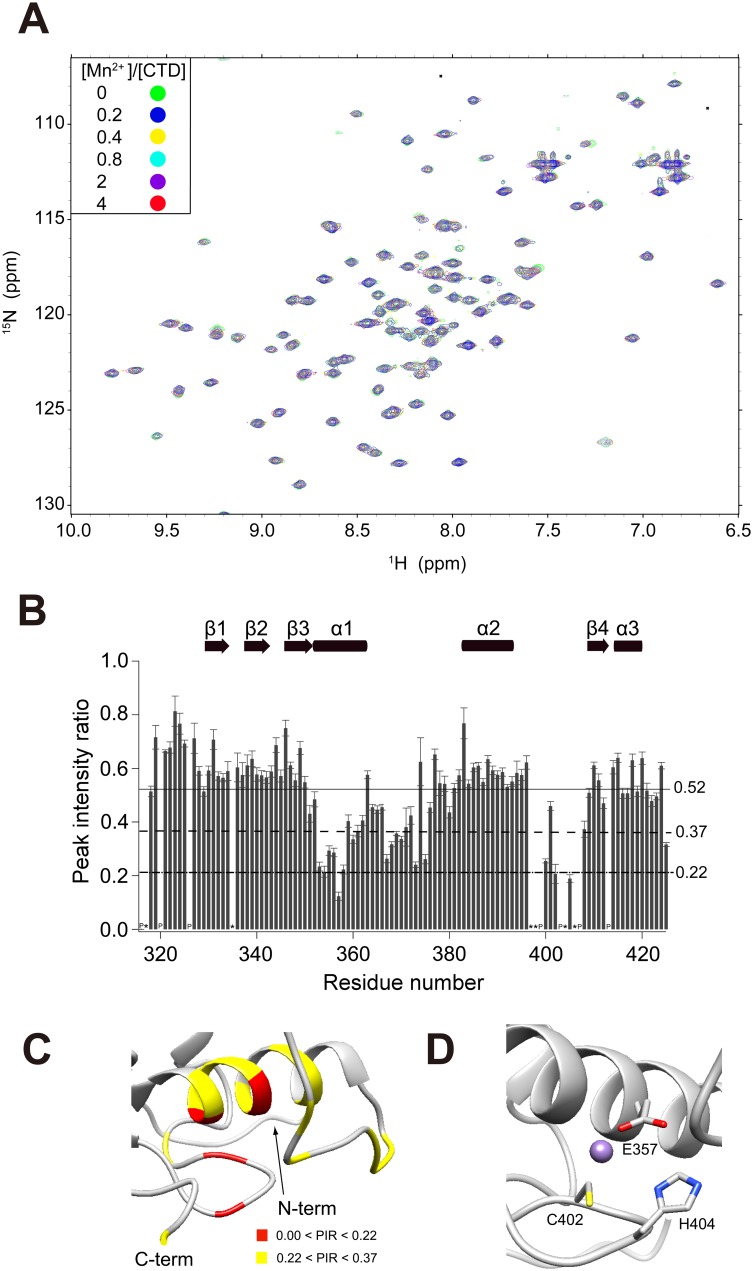
Characterization of interactions between aqMutL-CTD and Mn^2+^. (A) The ^1^H-^15^N HSQC spectra of 100 µM CTD at the various concentrations of Mn^2+^ are superimposed. (B) The peak intensity ratio of CTD in the absence and presence of 400 µM Mn^2+^ was plotted against the residue numbers. The upper solid and middle dashed lines represent ″mean value (0.52)″ and ″mean value − standard deviation (0.37)″. The bottom dotted line signifies ″mean value − 2× standard deviation (0.22)″. (C) Mn^2+^ binding sites based on the peak intensity ratio in B are shown with the color code: red, peak intensity ratio <0.22; yellow, 0.22< peak intensity ratio <0.37. (D) The binding sites for Mn^2+^ are magnified. The purple sphere indicates Mn^2+^.

NMR-based analysis was performed to examine the Mn^2+^ binding site of aqMutL-CTD at the residue level in solution. On the basis of the effect of the paramagnetic relaxation enhancement (PRE) of Mn^2+^, we expected to detect even transient and weak intermolecular interactions by observing a decrease in the peak intensity because PRE was previously shown to be proportional to the inverse sixth power of distance [29]. Although no significant perturbation was detected in the chemical or pseudo-contact shift (Figure 4A), marked decreases were observed in the NMR signals of CTD with the addition of Mn^2+^ (Figure 4B). Overall decreases in the peak intensity implied that the whole molecule of aqMutL-CTD was susceptible to the effects of the solvent PRE of Mn^2+^.

Large decreases in intensities were observed in several characteristic regions (peak intensity ratio <0.52): the region of α1 including the DQHA(X)_2_E(X)_4_ motif, the α1–α2 loop, and the α2-β4 loop including the CPHGRPI motif (Figures 4B and 4C). Among them, a marked decrease in the peak intensity ratio (<0.22) was observed in L354, E357, C402, and G405. Taken together with information on the characteristic coordination geometry of Mn^2+^ in the Mn^2+^-bound protein [30], we coordinated Mn^2+^ around E357, C402, and H404 to visualize Mn^2+^ binding sites (Figure 4D).

Far-UV CD measurements were performed to investigate the effects of Mn^2+^ binding on changes in the secondary structure and thermal stability (Figure 2). Although the far-UV CD spectrum was almost identical to that in the absence of metal ions (Figure 2A), the thermal transition curve was slightly different (Figure 2B). Mn^2+^ binding shifted the transition curve to slightly higher temperatures. These results implied that Mn^2+^ interacted less specifically and strongly with CTD than with Zn^2+^, which were consistent with the NMR results.

### Investigation of Intermolecular Interactions between ATP and aqMutL-CTD

**Figure 5:**
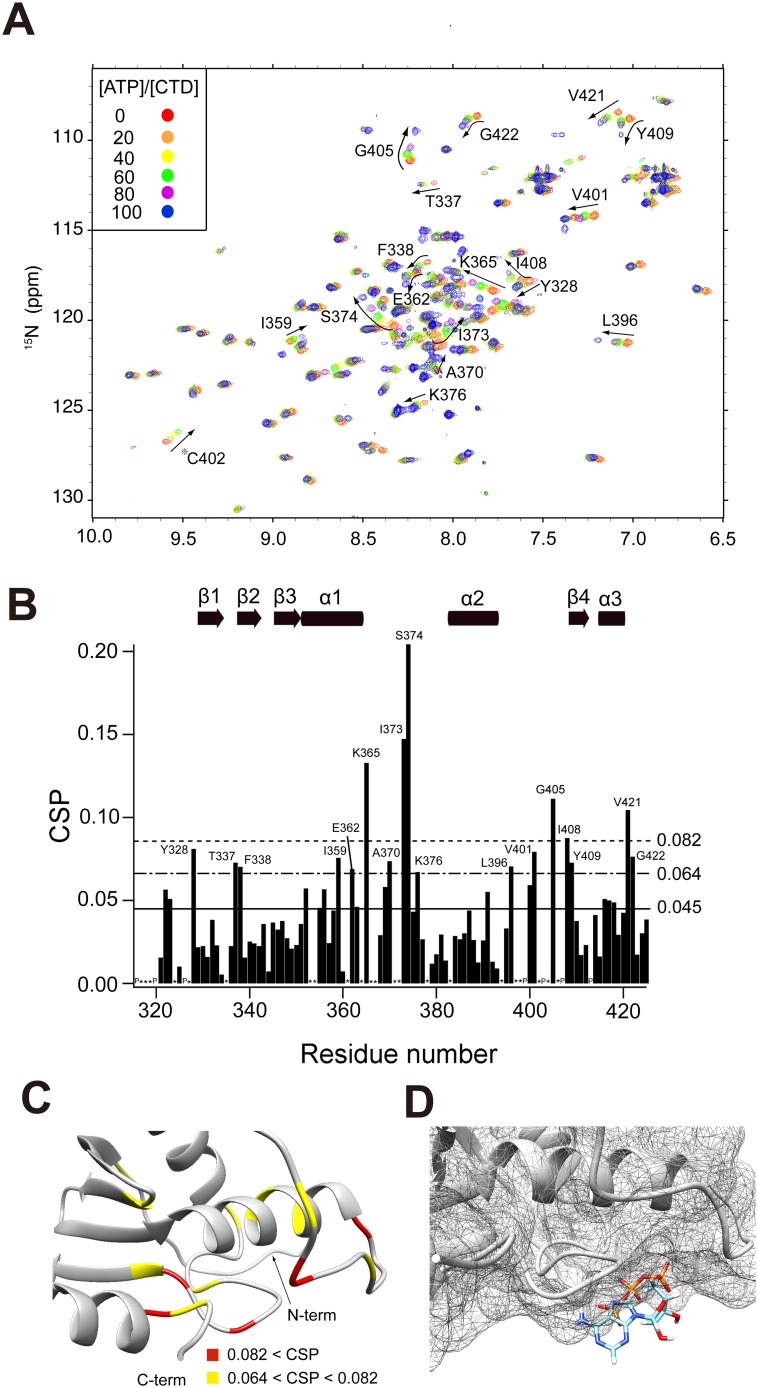
The characterization of interactions between aqMutL-CTD and ATP. (A) The ^1^H-^15^N HSQC spectra of 100 µM CTD at various concentrations of ATP are superimposed. The residues displaying significant perturbations are labeled using a one-letter amino acid code and residue numbers. Peaks of C402 (?) disappeared after [ATP]/[CTD] = 80 as the titration proceeded; it was then ruled out in the chemical shift perturbation (CSP) analysis. However, the label was displayed for the discussion below. (B) CSPs of the CTD in the presence of 8 mM ATP were plotted against residue numbers. The bottom solid line represents the ″mean value (0.045)″. The middle and upper dotted lines signify ″mean value+0.5× standard deviation (0.064)″ and ″mean value + standard deviation (0.082)″ for significant changes, respectively. (C) The degree of the perturbation was mapped onto the model structure with the color code: Red, CSP>0.082, yellow, 0.082>CSP>0.064. (D) The complex structure of the CTD and ATP as obtained by the docking simulation using MyPresto/Sievgene (see the Materials and Methods). The interacting sites are enlarged (right). ATP and aqMutL-CTD are represented by a ball and stick model and meshed surface model, respectively.

Although ATP binding to MutL is key to MMR, intermolecular interactions between the CTD and ATP remain largely unknown [11], [12], [14], [15], [31], [32]. Therefore, we obtained residue-based information on ATP binding using NMR spectroscopy. The titration of ATP against the CTD showed the shift of peaks with a fast exchange regime, which revealed the intermolecular interactions between the two molecules with weak affinity (Figure 5A). NMR chemical shift can be perturbed by the changes in electronic environments around NMR-active nuclei of interest. Therefore, several factors in the binding system induce perturbations of NMR chemical shifts such as the ligand binding without the conformational change and/or the ligand binding with the conformational change. It is often difficult to distinguish the mixed factors which are attributed to the apparent change in the chemical shift using only NMR data. Therefore, although we cannot assert the origin of a lot of chemical shift changes of aqMutL-CTD, ATP obviously binds to aqMutL-CTD and ATP-binding may induce structural rearrangements.

The detailed analysis for the affinity based on the chemical shift perturbation was hampered by the protein aggregation in the presence of the high ATP-concentration over 12 mM. It should be noted that examining of CTD aggregation is important to understand endonuclease activity of MutL-CTD. Therefore, we compared the intensity of CTD at each ATP concentration to examine the formation of aggregates. The results showed the comparable intensities: the NMR peak intensity of CTD in the presence of ATP with the 100-fold higher concentration than CTD did not decrease compared to the peak intensity of CTD without ATP, indicative of the absence of aggregation. This is because, in general, the formation of aggregates decreases significantly the intensity of the NMR signals. In order to obtain more evidence, we additionally performed the light scattering measurements of CTD in the absence and presence of ATP (Figure S9). High and low intensity of light scattering indicate large and insoluble aggregates as well as small and soluble proteins, respectively. For the reference intensity of aggregates, we also carried out the light scattering measurement of the amyloid fibril of Aβ_1–40_ peptides which is an ordered aggregate. As shown in Figure S9, the intensities of CTD in the absence and presence of ATP were much lower than those of the amyloid fibrils of Aβ_1–40_ peptides and were similar to the intensity of monomeric Aβ_1–40_ peptides. All of these data indicated the absence of aggregation of CTD in the presence of excess amounts of ATP but less than 12 mM.

Chemical shift perturbation analysis also revealed the binding sites for ATP. The residues in α1 (K365), the loop between α1 and α2 (I373 and S374), and the C-terminal parts (G405, I408, and V421) showed a high chemical shift perturbation (CSP) over 0.082 on ATP binding (Figure 5B). The perturbed residues were mapped on the model structure depending on the CSP values using the color codes (Figure 5C).

The docking simulation, which incorporated the CSP data, provided a putative scheme for the intermolecular interactions between the CTD and ATP. The results obtained indicated that ATP was bound to the cleft formed by α1 including the DQHA(X)_2_E(X)_4_E motif, the α1–α2 loop including the ACRISV motif, and the α2–β4 loop including the CPHGRPI motif (Figure 5D). The side chains of R358, K365, and N398 interacted electrostatically with the β-, α-, and γ-phosphates of ATP. The ribose and adenine of ATP were bound primarily beneath the α2–β4 loop.

The far-UV CD spectrum and thermal transition curve of CTD in the presence of ATP were identical to those without ATP, indicating that weak ATP binding did not change the secondary structure and global thermal stability (Figure 2).

Finally, to rule out the possibility of sharing binding sites for DNA, i.e., competitive binding between DNA and ATP, we directly measured the binding of DNA to aqMutL-CTD. As shown in Figure S6, excess amounts of DNA relative to aqMutL-CTD did not give any changes in the NMR signals of aqMutL-CTD, indicating that binding affinity of DNA to aqMutL-CTD is extremely low. Although a binding region for DNA was not determined experimentally, it is obvious that binding affinity of aqMutL-CTD for ATP is higher than that for DNA. As a result, if ATP shares a binding site with DNA, endonuclease activity of CTD should be affected by the presence of ATP.

However, the recent study by Shimada et al. demonstrated that Mn^2+^-dependent endonuclease activity of *Thermas thermophilus* MutL (ttMutL)-CTD did not depend on the presence of ATP. Endonuclease activity of ttMutL-CTD in the absence and presence showed same activity [13]. Moreover, Mn^2+^-dependent endonuclease activity of aqMutL-CTD was also not stimulated or inhibited by AMPPNP (Fukui K., unpublished data). These results indicate that ATP does not competitively bind to the dsDNA binding site of MutL-CTD with DNA, and vice versa.

Taken all together, the binding site on aqMutL-CTD for ATP which was revealed in this study is novel and is not used for the DNA binding (i.e., an active site of the nuclease).

### Investigation of ATP Binding to aqMutL-CTD

In order to obtain more information on binding specificity of ATP to aqMutL-CTD, we further carried out the NMR titration experiments on ADP binding to aqMutL-CTD (Figure S7) and the ITC measurements on both ATP and ADP binding to CTD, respectively (Figure S8).

A series of the NMR titration spectra of aqMutL-CTD with ADP revealed the large differences from those with ATP. Although the ADP titration spectra also showed the changes of NMR peaks, the number of perturbed peaks for ADP titration was less than that for ATP titration. Interestingly, all of the trajectories of the shifted peaks upon ADP titration including the peaks (e.g. G405, Y409, and G422) which showed the two different directions of the trajectory of the peak shift upon ATP titration were linear (Figure S7A). In addition, the magnitude of chemical shift perturbation on ADP titration was lower than that on ATP titration (Figure S7B). These results indicate that ADP also binds to aqMutL-CTD (Figure S7C) with the lower specificity and binding affinity than that for ATP binding.

At the same time, we can also exclude the possibility that perturbation of peaks by ATP and ADP titration was not induced by the ionic strength change of NMR solvent by addition of ATP and ADP. If there is an influence from the ionic strength of the added nucleotides, the shift of NMR peaks of aqMutL-CTD upon addition of ATP or ADP should show a similar pattern. However, as described above, the pattern of trajectory between ATP and ADP titration was obviously distinct, indicative of specific binding of ATP and ADP to aqMutL-CTD.

ITC results gave a further insight into ATP and ADP binding. The calorimetric titration showed endothermic reaction heat for titrating ATP or ADP to aqMutL-CTD (Figure S8). The magnitude of observed heat for ATP titration was greater than that for ADP titration by approximately five-fold, indicating more tight intermolecular interactions between ATP and aqMutL-CTD than those between ADP and CTD. Both titration was not saturated at the molar ratio ([nucleotide]/[aqMutL-CTD]) of even 25, consistent with the results of NMR titration. Most importantly, the endothermic peaks for both titration decreased gradually and the peaks of ATP titration were more rapidly decreased than those for ADP titration. Although the binding curves were too gentle to analyze the data precisely, these ITC data suggested that ATP binding is more specific than ADP binding with high binding affinity compared to ADP binding. The different binding affinity and specificity of ATP and ADP to MutL-CTD may come from a phosphate.

It should be noted that further binding assays using GTP, NTP, or dATP are required to obtain an insight into molecular origin on binding specificity of ATP to MutL-CTD.

## Discussion

We here demonstrated that Zn^2+^, Mn^2+^, and ATP bound to aqMutL-CTD without alternating the secondary structure. Only Zn^2+^ markedly increased thermal stability, and this has been attributed to changes in tertiary structures and/or dynamics. It should be noted that although the complete profile of thermal unfolding of aqMutL-CTD in the presence of Zn^2+^ was not obtained, it is obvious that cooperativity of thermal unfolding of Zn^2+^-bound aqMutL-CTD is higher than that of apo-CTD, indicating the increase in thermal stability. Many new NMR signals can be interpreted by reorganizing large parts of the tertiary structure (Figure 3A). Several key residues such as H353, E357, C371, C402, and H404 may be important for suppressing global motion due to the tight binding of Zn^2+^ (Figure 3B). Previous studies also reported that Zn^2+^ ions served as a structural factor rather than a catalytic contribution [33]–[35], implying a relationship between Zn^2+^ binding and stability. Since aqMutL-CTD functions at high temperatures above 90°C, Zn^2+^ binding should be essential for maintaining conformational stability for its function.

Mn^2+^ has been shown to play a pivotal role in the endonuclease activity of aqMutL-CTD. Although it has been suggested that the coordination number and residue composition of the Zn^2+^-binding site of MutL may preclude Mn^2+^ binding based on the bioinformatics of metal-binding proteins [35]–[40], we clarified that the Mn^2+^ binding sites of aqMutL-CTD were located around the endonuclease activity sites between the two motifs, DQHA(X)_2_E(X)_4_E and CPHGRPI (Figure 4). The reported endonuclease activity assay of the aqMutL-CTD mutants (E357K and H404A) displayed the inactivation of the Mn^2+^-dependent incision of supercoiled DNA, which may have been due to decreases in the binding capability of the CTD for Mn^2+^ [41]. These findings support the results of the present study in which E357 and H404 consisted of binding sites for Mn^2+^, as shown in Figure 4B.

Mn^2+^ shared CTD binding sites with Zn^2+^, with similar coordination geometries (Figures 3 and 4). Because Zn^2+^ enhanced the endonuclease activity of bsMutL in the presence of Mn^2+^ [1], these two divalent metal ions may be coordinated together for MMR *in vivo* to ensure both stability and function; however, detailed future work is required to clarify these intermolecular interactions.

ATP binding to MutL and MutLα for MMR has been demonstrated and the NTD was predominantly suggested to accommodate ATP [14], [31]. However, there has been little focus on ATP binding to the CTD and no consensus on their intermolecular interactions. Mauris J. *et al.* showed that ATP did not bind aqMutL-CTD using the filter binding assay and further indicated the absence of ATP binding motif in aqMutL-CTD [41]. In contrast, it could be implied that the intermolecular interactions between CTD from ttMutL and ATP may be possible [42]. This discrepancy may reflect the difficulty in detecting weak intermolecular interactions, which are markedly susceptible to subtle changes in the surrounding conditions used such as temperature, salt concentration, and pH.

We here demonstrated weak and specific interactions between ATP and aqMutL-CTD using solution-state NMR at the residue level and ITC at the molecular level. By incrementally adding excess ATP to aqMutL-CTD, we revealed gradual shifts in peak positions, which indicated weak intermolecular interactions. The linear shift followed successively by a linear shift in a different way, as represented by G405, I408, and Y409 (Figure 5), implied the two states of the ATP-bound CTD. Considering the concentrations of ATP and CTD used here, the affinity of the CTD for ATP was markedly lower than that of the NTD, the dissociation constant of which is approximately 20 nM (Fukui K., unpublished data). ITC data also showed the weak affinity of CTD for ATP, consistent with the NMR results.

Chemical shift perturbation analysis and the docking simulation allowed us to determine the binding sites for ATP, which included the motifs conserved in the various species (Figure S5), and implied that the binding of ATP to the CTD may be a common feature. The feature of the rigid conformations of the α2–β4 loop containing the conserved CPHGRPI motif due to hydrogen bond networks (Figure 1D) may be involved in the effective binding of metal ions and ATP. Interestingly, the ATP binding sites of aqMutL-CTD were included in the regions responsible for the interdomain interactions for the NTD, which have been suggested to promote the endonuclease activity of the CTD [12], [13], [15]. This indicates the possibility of regulatory role of ATP binding to CTD in CTD-NTD interdomain interaction.

In addition, Fukui K. and coworkers modified Cys496 of ttMutL-CTD, which is the conserved cysteinyl residue in CPHGRPI motif corresponding to Cys402 of aqMutL-CTD [42]. They showed that chemical modification of Cys496 with DTNB and the substitution of Cys496 with an Ala residue in ttMutL caused the decrease in the efficiency of AMPPNP-dependent suppression of endonuclease activity. Considering that Cys402 of aqMutL-CTD was located in the ATP binding sites (Figure 5) which existed in the interface for CTD-NTD interdomain interaction as mentioned above, these assays indicated the high possibility that ATP binding to MutL-CTD was involved in the ATP-dependent suppression of endonuclease activity through suppressing CTD-NTD interdomain interaction.

A previous study demonstrated that the Mn^2+^-dependent nonspecific endonuclease activity of bsMutL was inhibited at relatively high ATP concentrations and enhanced at low ATP concentrations [1]. It was also reported that endonuclease activity of aqMutL and ttMutL at high and low concentrations were enhanced and inhibited in the presence of ATP and Mn^2+^, respectively [13], [41]. Although these apparently contradictory results have hampered the establishment of a structural model for the regulation of MutL endonuclease activity both *in vitro* and *in vivo*, the relative concentrations between the CTD and ATP are more likely to be essential for more clearly understanding a mechanistic model of MMR *in vitro*.

Accordingly, binding of ATP to an endonuclease domain of MutL and its involvement in interfering with the CTD-NTD interdomain interaction is one of the missing keys for a mechanistic model for MMR.

**Figure 6:**
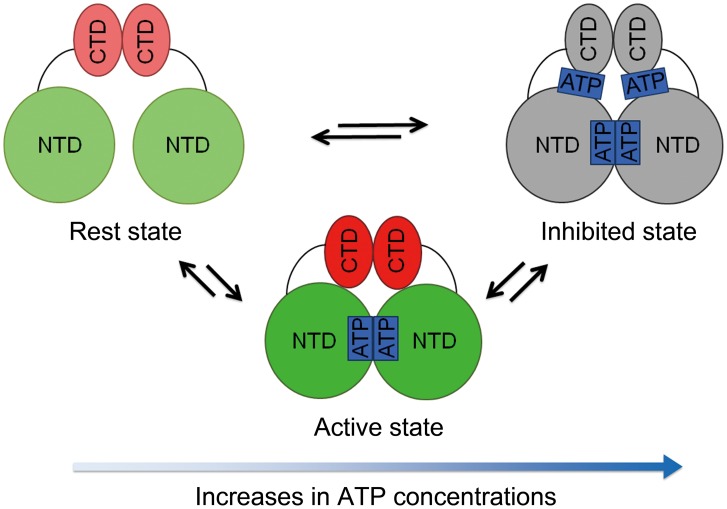
Activation-attenuation model for the ATP concentration-dependent response of MutL endonuclease activity. The three conformational states of MutL (rest, active, and inhibited states) are in equilibrium. The dominant conformational state can be shifted depending on the differences in the relative concentration between MutL and ATP. The rest state of MutL in the absence of ATP (left) is predominantly populated. The small increase in the ATP concentration drives the ATP binding to NTD thereby stabilizing the active state (middle). At the high ATP concentration, however, the inhibited state which suppresses endonuclease activity is favored by accommodating further ATP to CTD (right). It should be noted that the complicated conformational states of ATP-bound CTD which are ATP concentration-dependent are not considered for simplicity. The ellipses and circles indicate the CTD and NTD, respectively. Blue rectangles show ATP.

Based on the reported and current results, we proposed the ATP concentration-dependent response of MutL endonuclease activity, “activation-attenuation model” on the basis of the equilibrium among the three conformational states (rest state, active state, and inhibited state) (Figure 6). In the absence of ATP, the rest state predominantly exists thus there are no interdomain interactions. On the one hand, since the increase in the ATP concentration with the fixed MutL concentration shifts an equilibrium from the rest state to the ATP-bound states, the active state, where the interdomain interaction is prevailed by the ATP binding to NTD due to its higher binding affinity for NTD than CTD, is stabilized. Further increasing of the ATP concentration mostly accumulates the inhibited state since excess amounts of ATP promote the ATP binding to CTD which disfavors the active interdomain interaction. These hampered interdomain interactions on the increases in the ATP concentration in the cell in turn may suppress the endonuclease activity of MutL. It should be noted that the decrease in endonuclease activity at the high ATP concentration is not attributed to the formation of aggregates of CTD and there is no competitive binding between ATP and DNA as we described in detail.

Because the concentration of ATP in a bacterial cell is known to be approximately 10 mM [43] and the intracellular concentration of aqMutL in a thermophilic bacterium is approximately 300 nM [13], the endonuclease activity of aqMutL is expected to be suppressed by ATP *in vivo*. This suppressing effect on the non-specific degradation of DNA resulting from the lack of specificity may be effective for MutL endonuclease activity. The ATP-dependent suppression of MutL endonuclease activity may be released when MutL is recruited to the MutS:mismatch complex to initiate downstream repair machinery. Hence, binding of MutL to the MutS:mismatch complex unlocks the ATP-dependent inhibition of its non-specific endonuclease activity.

Although further detailed studies on the model suggested are required with quantitative values, experimental evidence on ATP binding to an endonuclease domain of MutL and the binding site described here will be helpful to understand a mechanistic model for the ATP-dependent regulation of MutL in MMR.

## Supporting Information

Figure S1 **Effect of the buffer on the ^1^H-^15^N HSQC spectrum of aqMutL-CTD.** The two spectra of aqMutL-CTD samples in potassium phosphate buffer (green) and Tris-HCl buffer (black) were superimposed. The two buffers containing 100 mM KCl, 5 mM DTT, and 1 mM EDTA were kept at the same pH value (pH 7.0) at 313 K. Several peaks in Tris-HCl buffer were severely broadened (Q319, Y324, T327, H353, E367, C371, G378, and E383). Some peaks were shifted slightly (E329, K365, V375, K376, K380, and C402).
(PDF)

Figure S2 **The ^1^H-^15^N HSQC spectra of His-specific labeled aqMutL-CTD.** The ^1^H-^15^N HSQC spectrum of the His-specific labeled CTD (green) was superimposed onto that of the uniformly ^15^N-labeled CTD sample (gray) acquired at 313 K in 50 mM potassium phosphate buffer (pH 6.8) containing 100 mM KCl, 5 mM DTT, and 1 mM EDTA.
(PDF)

Figure S3 **The ^1^H-^15^N HSQC spectra of Arg and Lys-specific labeled aqMutL-CTD.** (A) The ^1^H-^15^N HSQC spectrum of the Arg-specific inversely-labeled CTD (gray) was superimposed onto that of the uniformly ^15^N-labeled CTD sample (green) acquired at 313 K in 50 mM potassium phosphate buffer (pH 6.8) containing 100 mM KCl, 5 mM DTT, and 1 mM EDTA. (B) The ^1^H-^15^N HSQC spectrum of the Lys-specific inversely-labeled CTD (gray) was also superimposed onto that of the uniformly ^15^N-labeled CTD (green) acquired at 313 K in 50 mM potassium phosphate buffer (pH 6.8) containing 100 mM KCl, 5 mM DTT, and 1 mM EDTA.
(PDF)

Figure S4 **Summary of the parameters obtained from the relaxation measurements of aqMutL-CTD.** The longitudinal (*R*_1_) (A) and transverse (*R*_2_) relaxation rates (B) and ^1^H-^15^N NOE values (C) were plotted against the residue numbers. Secondary structures obtained from TALOS+ were depicted at the top of the panels.
(PDF)

Figure S5 **Multiple sequence alignments of MutL-CTD.** The sequences of the CTDs of MutH-less species (bsMutL, ngMutL, aqMutL, and human MutL homolog PMS2) were displayed using ClustalW (http://clustalw.ddbj.nig.ac.jp). Conserved motifs were highlighted by rectangles. The residue numbers of aqMutL-CTD (K365 and D366) were displayed to note the missing primary sequences of aqMutL-CTD among MutL-homologs. ″*″ indicates perfectly matched residues.
(PDF)

Figure S6 **The ^1^H-^15^N HSQC spectra of aqMutL-CTD with and without dsDNA.** The ^1^H-^15^N HSQC spectra of 100 µM aqMutL-CTD at the various dsDNA concentrations were obtained at 313 K in 25 mM Tris-HCl buffer (pH 7.0) containing 25 mM KCl, 5 mM DTT, and 1 mM EDTA. The spectra were overlaid for comparison.
(PDF)

Figure S7 **The characterization of interactions between aqMutL-CTD and ADP.** (A) The ^1^H-^15^N HSQC spectra of 100 µM CTD at various concentrations of ADP are superimposed. The residues displaying significant perturbations are labeled using a one-letter amino acid code and residue numbers. (B) CSPs of the CTD in the presence of 8 mM ADP were plotted against residue numbers. The bottom solid line represents the ″mean value (0.027)″. The middle and upper dotted lines signify ″mean value+0.5× standard deviation (0.044)″ and ″mean value + standard deviation (0.062)″ for significant changes, respectively. (C) The degree of the perturbation was mapped onto the model structure with the color code: Red, CSP>0.062, yellow, 0.062>CSP>0.044.
(PDF)

Figure S8 **Calorimetric titration of ATP and ADP to aqMutL-CTD.** ATP (A) and ADP (B) in the syringe were titrated to CTD in the reaction cell, respectively. Thermograms and binding isotherms are shown in the upper and lower panels, respectively. The corresponding heat of dilution of ATP or ADP titrated to the buffer was used to correct the data. Solid lines to guide the eye in the lower panels are presented.
(PDF)

Figure S9 **Light scattering measurements of CTD.** The normalized intensity of light scattering of CTD solution in the absence (−) and presence (+) of ATP is shown. Light scattering of the monomeric Aβ_1–40_ peptides and the Aβ_1–40_ amyloid fibrils of Aβ_1–40_ peptides which are aggregates is also displayed for comparison. The concentrations of CTD, ATP, and Aβ_1–40_ peptides were, 16, 160, and 30 µM, respectively.
(PDF)

Figure S10 **Superimposition of homology-modeled structures of aqMutL-CTD with the various template X-ray structures.** The model structure generated by using the template structures of bsMutL-CTD with NMR secondary structure constraints is colored by gray. The model structures templated by using bsMutL-CTD (blue), NgoL MutL-CTD (pink) and PMS1-CTD (green) without NMR constrains are also shown with the monomeric structures.
(PDF)

## References

[1] Pillon MC, Lorenowicz JJ, Uckelmann M, Klocko AD, Mitchell RR, et al (2010) Structure of the Endonuclease Domain of MutL: Unlicensed to Cut. Mol Cell 39: 145–151 10.1016/j.molcel.2010.06.027 20603082 PMC2933357

[2] Fishel R, Lescoe MK, Rao MR, Copeland NG, Jenkins NA, et al (1993) The human mutator gene homolog MSH2 and its association with hereditary nonpolyposis colon cancer. Cell 75: 1027–1038 10.1016/0092-8674(93)90546-3 8252616

[3] Fishel R, Kolodner RD (1995) Identification of mismatch repair genes and their role in the development of cancer. Curr Opin Genet Dev 5: 382–395 10.1016/0959-437X(95)80055-7 7549435

[4] Larrea AA, Lujan SA, Kunkel TA (2010) SnapShot: DNA Mismatch Repair. Cell 141: 730–730.e731 10.1016/j.cell.2010.05.002 20478261

[5] Iyer RR, Pluciennik A, Burdett V, Modrich PL (2006) DNA Mismatch Repair: Functions and Mechanisms. Chem Rev 106: 302–323 10.1021/cr0404794 16464007

[6] Fukui K (2010) DNA Mismatch Repair in Eukaryotes and Bacteria. J Nucleic Acids 2010: 1–16 10.1016/j.bbapap.2010.01.017 PMC2915661 20725617

[7] Modrich P (2006) Mechanisms in Eukaryotic Mismatch Repair. J Biol Chem 281: 30305–30309 10.1074/jbc.R600022200 16905530 PMC2234602

[8] Kadyrov FA, Dzantiev L, Constantin N, Modrich P (2006) Endonucleolytic Function of MutLα in Human Mismatch Repair. Cell 126: 297–308 10.1016/j.cell.2006.05.039 16873062

[9] Kadyrov FA, Holmes SF, Arana ME, Lukianova OA, O’Donnell M, et al (2007) Saccharomyces cerevisiae MutL Is a Mismatch Repair Endonuclease. J Biol Chem 282: 37181–37190 10.1074/jbc.M707617200 17951253 PMC2302834

[10] Guarné A, Ramon-Maiques S, Wolff EM, Ghirlando R, Hu X, et al (2004) Structure of the MutL C-terminal domain: a model of intact MutL and its roles in mismatch repair. EMBO J 23: 4134–4145 10.1038/sj.emboj.7600412 15470502 PMC524388

[11] Sacho EJ, Kadyrov FA, Modrich P, Kunkel TA, Erie DA (2008) Direct Visualization of Asymmetric Adenine Nucleotide-Induced Conformational Changes in MutLα. Mol Cell 29: 112–121 10.1016/j.molcel.2007.10.030 18206974 PMC2820111

[12] Iino H, Kim K, Shimada A, Masui R, Kuramitsu S, et al (2011) Characterization of C- and N-terminal domains of Aquifex aeolicusMutL endonuclease: N-terminal domain stimulates the endonuclease activity of C-terminal domain in a zinc-dependent manner. Biosci Rep 31: 309–322 10.1042/BSR20100116 20961292

[13] Shimada A, Kawasoe Y, Hata Y, Takahashi TS, Masui R, et al (2013) MutS stimulates the endonuclease activity of MutL in an ATP-hydrolysis-dependent manner. FEBS J 280: 3467–3479 10.1111/febs.12344 23679952

[14] Ban C, Yang W (1998) Crystal structure and ATPase activity of MutL: implications for DNA repair and mutagenesis. Cell 95: 541–552 10.1016/S0092-8674(00)81621-9 9827806

[15] Yamamoto T, Iino H, Kim K, Kuramitsu S, Fukui K (2011) Evidence for ATP-dependent structural rearrangement of nuclease catalytic site in DNA mismatch repair endonuclease MutL. J Biol Chem 286: 42337–42348 10.1074/jbc.M111.277335 21953455 PMC3234979

[16] Namadurai S, Jain D, Kulkarni DS, Tabib CR, Friedhoff P, et al (2010) The C-Terminal Domain of the MutL Homolog from Neisseria gonorrhoeae Forms an Inverted Homodimer. PLoS ONE 5: e13726 10.1371/journal.pone.0013726.t001 21060849 PMC2965676

[17] Gueneau E, Dherin C, Legrand P, Tellier-Lebegue C, Gilquin B, et al (2013) Structure of the MutLα C-terminal domain reveals how Mlh1 contributes to Pms1 endonuclease site. Nat Struct Mol Biol 20: 461–468 10.1038/nsmb.2511 23435383

[18] Kuramitsu S, Hiromi K, Hayashi H, Morino Y, Kagamiyama H (1990) Pre-steady-state kinetics of Escherichia coli aspartate aminotransferase catalyzed reactions and thermodynamic aspects of its substrate specificity. Biochemistry 29: 5469–5476. 2201406 10.1021/bi00475a010

[19] Delaglio F, Grzesiek S, Vuister G, Zhu G, Pfeifer J, et al (1995) NMRPipe: A multidimensional spectral processing system based on UNIX pipes. J Biomol NMR 6: 277–293 10.1007/BF00197809 8520220

[20] Dosset P, Hus JC, Blackledge M, Marion D (2000) Efficient analysis of macromolecular rotational diffusion from heteronuclear relaxation data. J Biomol NMR 16: 23–28 10.1023/A:1008305808620 10718609

[21] Shekhtman A, Burz DS (2011) Protein NMR Techniques. Humana Press. 1 pp.

[22] Eswar N, Webb B, Marti-Renom MA, Madhusudhan MS, Eramian D, et al.. (2001) Comparative Protein Structure Modeling Using MODELLER. Coligan JE, Dunn BM, Speicher DW, Wingfield PT, editors Hoboken, NJ, USA: John Wiley & Sons, Inc. doi:10.1002/0471140864.ps0209s50.

[23] Guex N, Peitsch MC (1997) SWISS-MODEL and the Swiss-Pdb Viewer: An environment for comparative protein modeling. Electrophoresis 18: 2714–2723 10.1002/elps.1150181505 9504803

[24] Fukunishi Y, Mikami Y, Nakamura H (2005) Similarities among receptor pockets and among compounds: analysis and application to in silico ligand screening. J Mol Graph Model 24: 34–45 10.1016/j.jmgm.2005.04.004 15950507

[25] Wang J, Cieplak P, Kollman PA (2000) How well does a restrained electrostatic potential (RESP) model perform in calculating conformational energies of organic and biological molecules? J Comput Chem 21: 1049–1074 doi:;10.1002/1096-987X(200009)21: 12<1049::AID-JCC3>3.0.CO;2-F

[26] Lee YH, Chatani E, Sasahara K, Naiki H, Goto Y (2009) A comprehensive model for packing and hydration for amyloid fibrils of beta2-microglobulin. J Biol Chem 284(4): 2169–75 10.1074/jbc.M806939200 19017634

[27] Levitt M (2009) Nature of the protein universe. Proc Natl Acad Sci USA 106: 11079–11084 10.1073/pnas.0905029106 19541617 PMC2698892

[28] Shen Y, Delaglio F, Cornilescu G, Bax A (2009) TALOS+: a hybrid method for predicting protein backbone torsion angles from NMR chemical shifts. J Biomol NMR 44: 213–223 10.1007/s10858-009-9333-z 19548092 PMC2726990

[29] Vega-Rocha S, Byeon I-JL, Gronenborn B, Gronenborn AM, Campos-Olivas R (2007) Solution structure, divalent metal and DNA binding of the endonuclease domain from the replication initiation protein from porcine circovirus 2. J Mol Biol 367: 473–487 10.1016/j.jmb.2007.01.002 17275023

[30] Shumilin IA, Bauerle R, Kretsinger RH (2003) The High-Resolution Structure of 3-Deoxy- d- arabino-heptulosonate-7-phosphate Synthase Reveals a Twist in the Plane of Bound Phosphoenolpyruvate. Biochemistry 42: 3766–3776 10.1021/bi027257p 12667068

[31] Guarné A, Junop MS, Yang W (2001) Structure and function of the N-terminal 40 kDa fragment of human PMS2: a monomeric GHL ATPase. EMBO J 20: 5521–5531 10.1093/emboj/20.19.5521 11574484 PMC125661

[32] Ban C, Junop M, Yang W (1999) Transformation of MutL by ATP binding and hydrolysis: a switch in DNA mismatch repair. Cell 97: 85–97 10.1016/S0092-8674(00)80717-5 10199405

[33] Ryde U (1999) Carboxylate binding modes in zinc proteins: a theoretical study. Biophys J 77: 2777–2787 10.1016/S0006-3495(99)77110-9 10545376 PMC1300550

[34] Karlin S, Zhu ZY (1997) Classification of mononuclear zinc metal sites in protein structures. Proc Natl Acad Sci USA 94: 14231–14236. 9405595 10.1073/pnas.94.26.14231 PMC24919

[35] Guarné A (2012) Chapter 3 - The Functions of MutL in Mismatch Repair: The Power of Multitasking. In: Doetsch PW, editor. Progress in Molecular Biology and Translational Science. Mechanisms of DNA Repair. Academic Press, Vol. 110: 41–70 10.1016/B978-0-12-387665-2.00003-1 22749142

[36] Dudev M, Wang J, Dudev T, Lim C (2006) Factors governing the metal coordination number in metal complexes from Cambridge Structural Database analyses. J Phys Chem B 110: 1889–1895 10.1021/jp054975n 16471760

[37] Dudev T, Lim C (2003) Principles governing Mg, Ca, and Zn binding and selectivity in proteins. Chem Rev 103: 773–788 10.1021/cr020467n 12630852

[38] Dudev T, Lin Y-L, Dudev M, Lim C (2003) First-second shell interactions in metal binding sites in proteins: a PDB survey and DFT/CDM calculations. J Am Chem Soc 125: 3168–3180 10.1021/ja0209722 12617685

[39] Harding MM (2004) The architecture of metal coordination groups in proteins. Acta Crystallogr D Biol Crystallogr 60: 849–859 10.1107/S0907444904004081 15103130

[40] Harding MM (2006) Small revisions to predicted distances around metal sites in proteins. Acta Crystallogr D Biol Crystallogr 62: 678–682 10.1107/S0907444906014594 16699196

[41] Mauris J, Evans TC (2009) Adenosine triphosphate stimulates Aquifex aeolicus MutL endonuclease activity. PLoS ONE 4: e7175 10.1371/journal.pone.0007175 19777055 PMC2744016

[42] Fukui K, Nishida M, Nakagawa N, Masui S (2008) Bound nucleotide controls the endonuclease activity of mismatch repair enzyme MutL. J Biol Chem 283(18): 12136–45 10.1074/jbc.M800110200 18310077

[43] Bennett BD, Kimball EH, Gao M, Osterhout R, Van Dien SJ, et al (2009) Absolute metabolite concentrations and implied enzyme active site occupancy in Escherichia coli. Nat Chem Biol 5: 593–599 10.1038/nchembio.186 19561621 PMC2754216

