# Supplementary figures and images for: NMR Characterization of the Interaction of the Endonuclease Domain of MutL with Divalent Metal Ions and ATP
Source: PLoS One. 2014 Jun 5;9(6):e98554. doi: 10.1371/journal.pone.0098554 (PMC4047009; doi:10.1371/journal.pone.0098554)

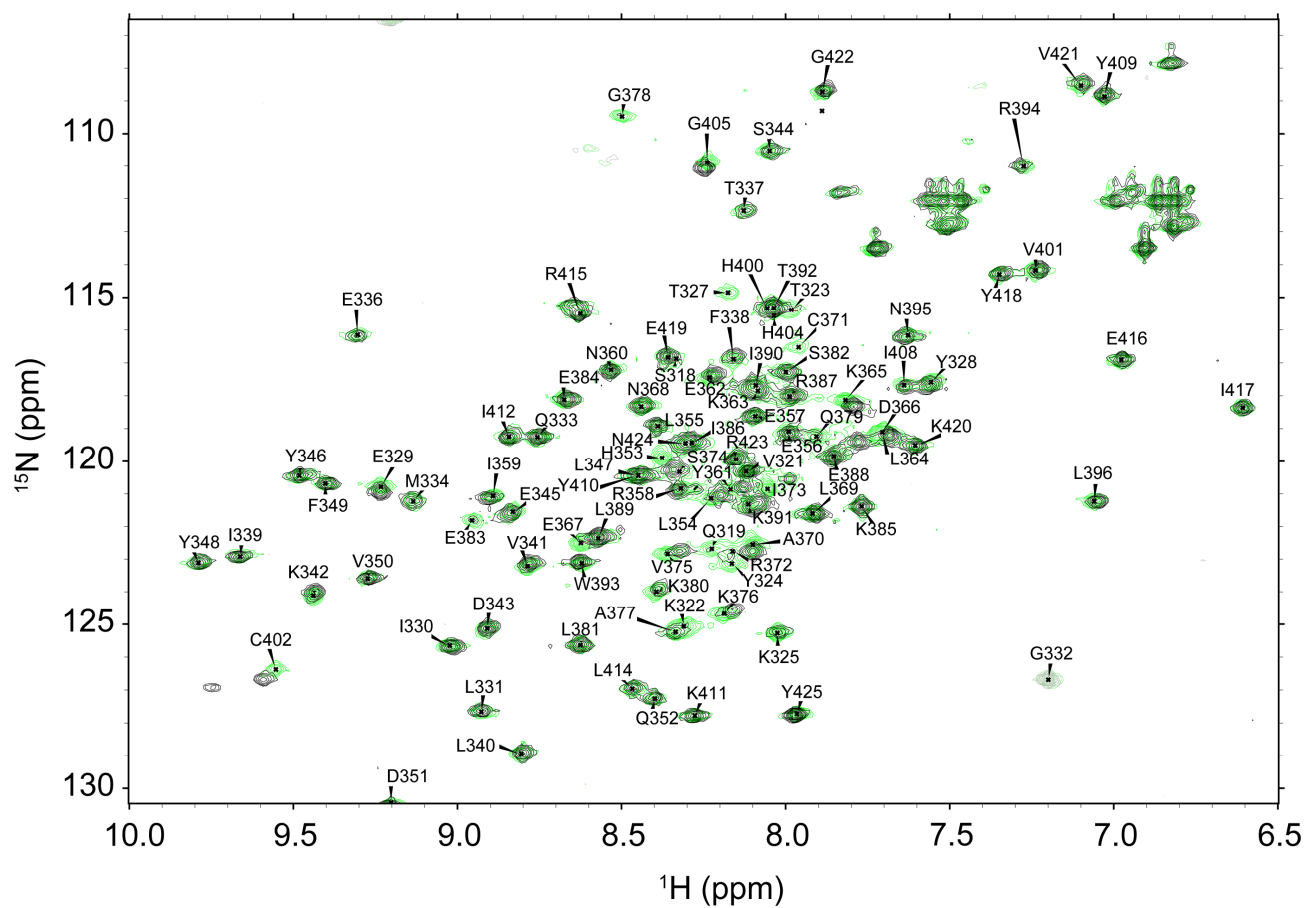

Supplement: Figure S1 — Effect of the buffer on the 1H-15N HSQC spectrum of aqMutL-CTD. The two spectra of aqMutL-CTD samples in potassium phosphate buffer (green) and Tris-HCl buffer (black) were superimposed. The two buffers containing 100 mM KCl, 5 mM DTT, and 1 mM EDTA were kept at the same pH value (pH 7.0) at 313 K. Several peaks in Tris-HCl buffer were severely broadened (Q319, Y324, T327, H353, E367, C371, G378, and E383). Some peaks were shifted slightly (E329, K365, V375, K376, K380, and C402). (PDF) [file pone.0098554.s001.pdf]

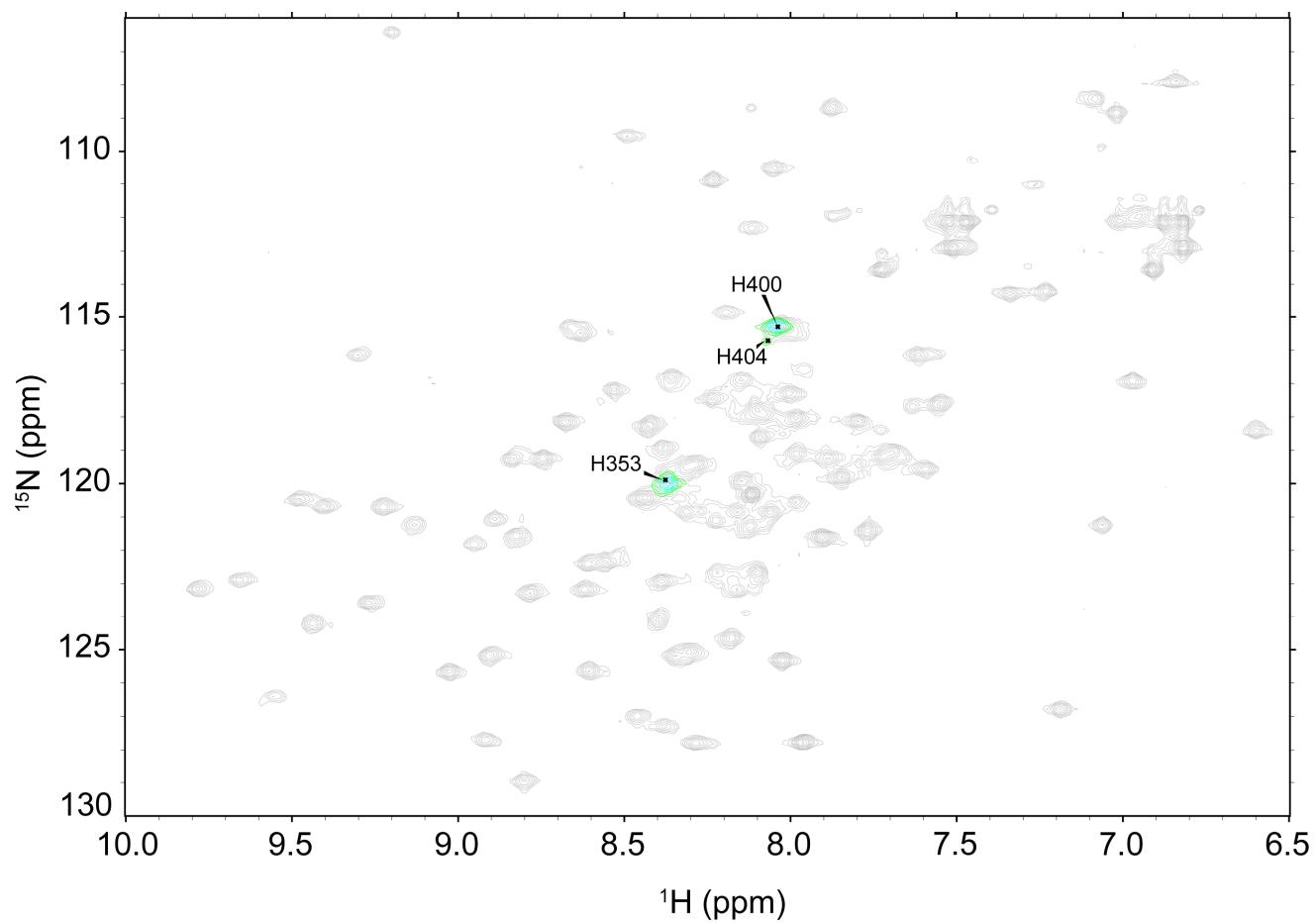

Supplement: Figure S2 — The 1H-15N HSQC spectra of His-specific labeled aqMutL-CTD. The 1H-15N HSQC spectrum of the His-specific labeled CTD (green) was superimposed onto that of the uniformly 15N-labeled CTD sample (gray) acquired at 313 K in 50 mM potassium phosphate buffer (pH 6.8) containing 100 mM KCl, 5 mM DTT, and 1 mM EDTA. (PDF) [file pone.0098554.s002.pdf]

**A**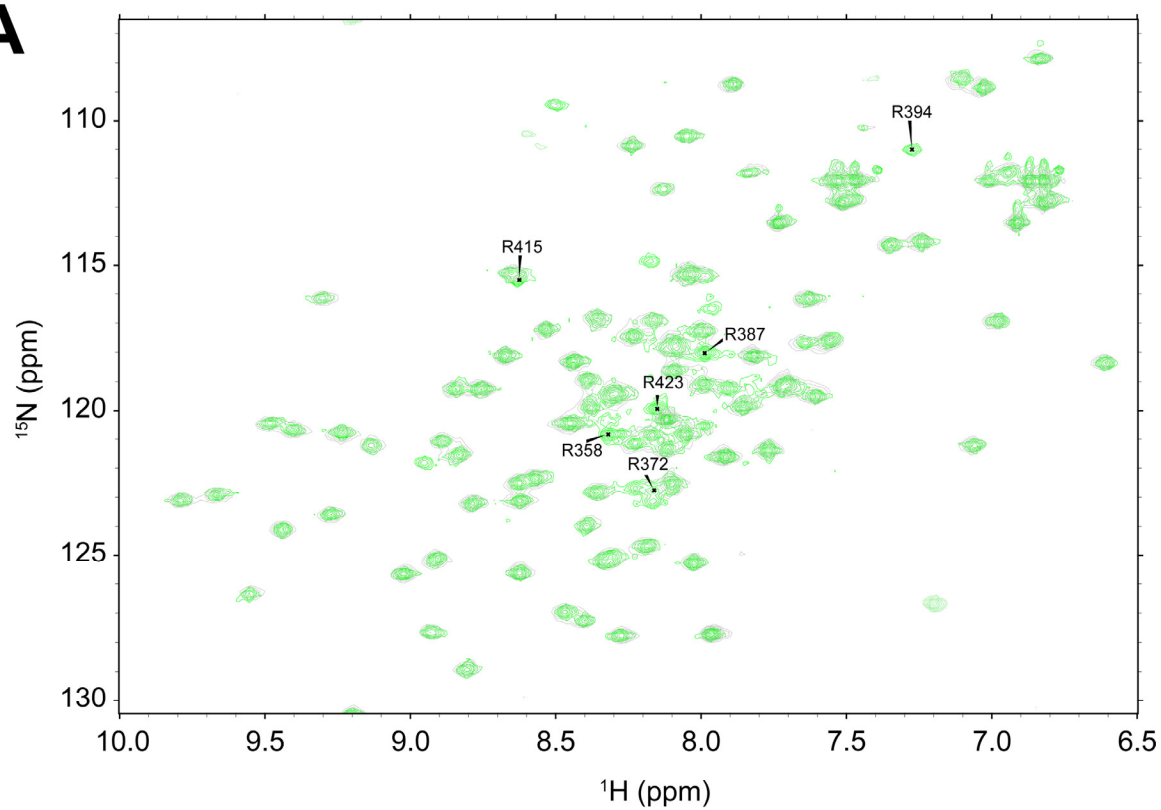**B**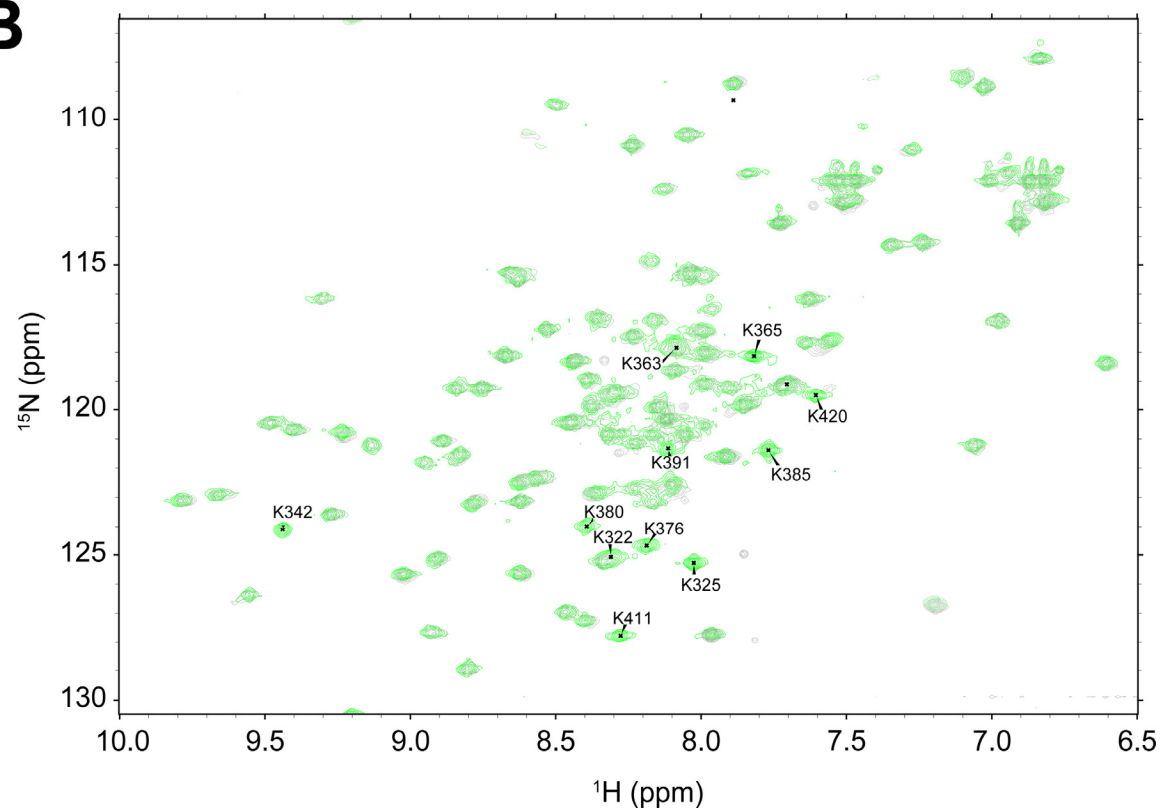

Supplement: Figure S3 — The 1H-15N HSQC spectra of Arg and Lys-specific labeled aqMutL-CTD. (A) The 1H-15N HSQC spectrum of the Arg-specific inversely-labeled CTD (gray) was superimposed onto that of the uniformly 15N-labeled CTD sample (green) acquired at 313 K in 50 mM potassium phosphate buffer (pH 6.8) containing 100 mM KCl, 5 mM DTT, and 1 mM EDTA. (B) The 1H-15N HSQC spectrum of the Lys-specific inversely-labeled CTD (gray) was also superimposed onto that of the uniformly 15N-labeled CTD (green) acquired at 313 K in 50 mM potassium phosphate buffer (pH 6.8) containing 100 mM KCl, 5 mM DTT, and 1 mM EDTA. (PDF) [file pone.0098554.s003.pdf]

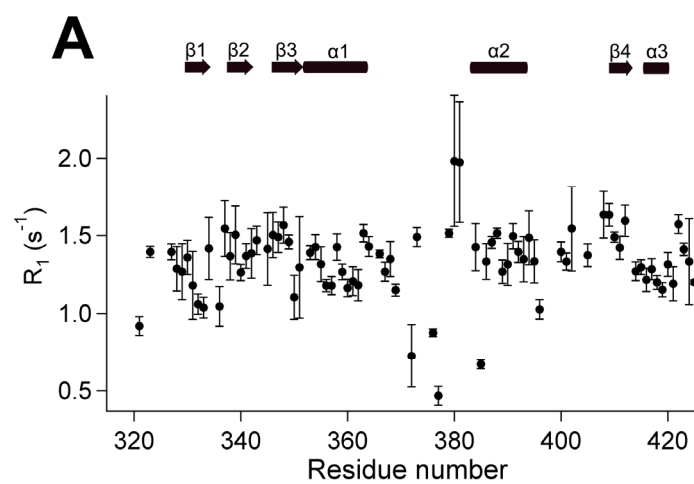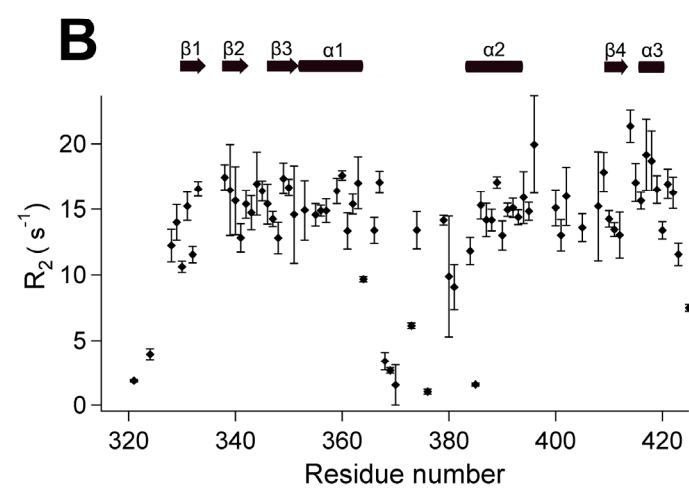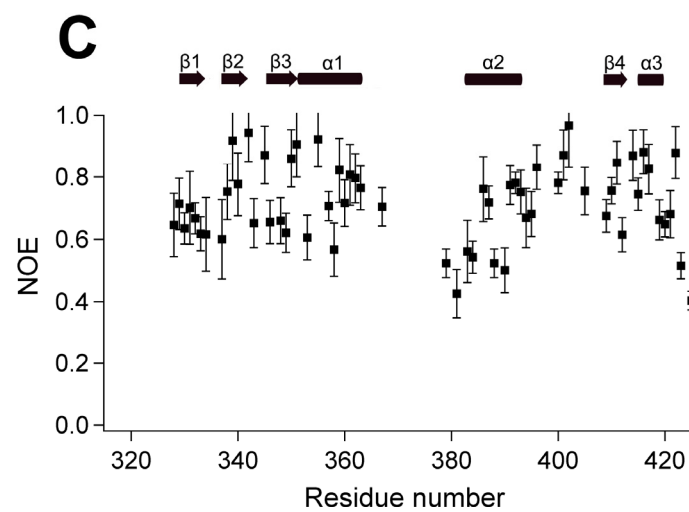

Supplement: Figure S4 — Summary of the parameters obtained from the relaxation measurements of aqMutL-CTD. The longitudinal (R 1) (A) and transverse (R 2) relaxation rates (B) and 1H-15N NOE values (C) were plotted against the residue numbers. Secondary structures obtained from TALOS+ were depicted at the top of the panels. (PDF) [file pone.0098554.s004.pdf]

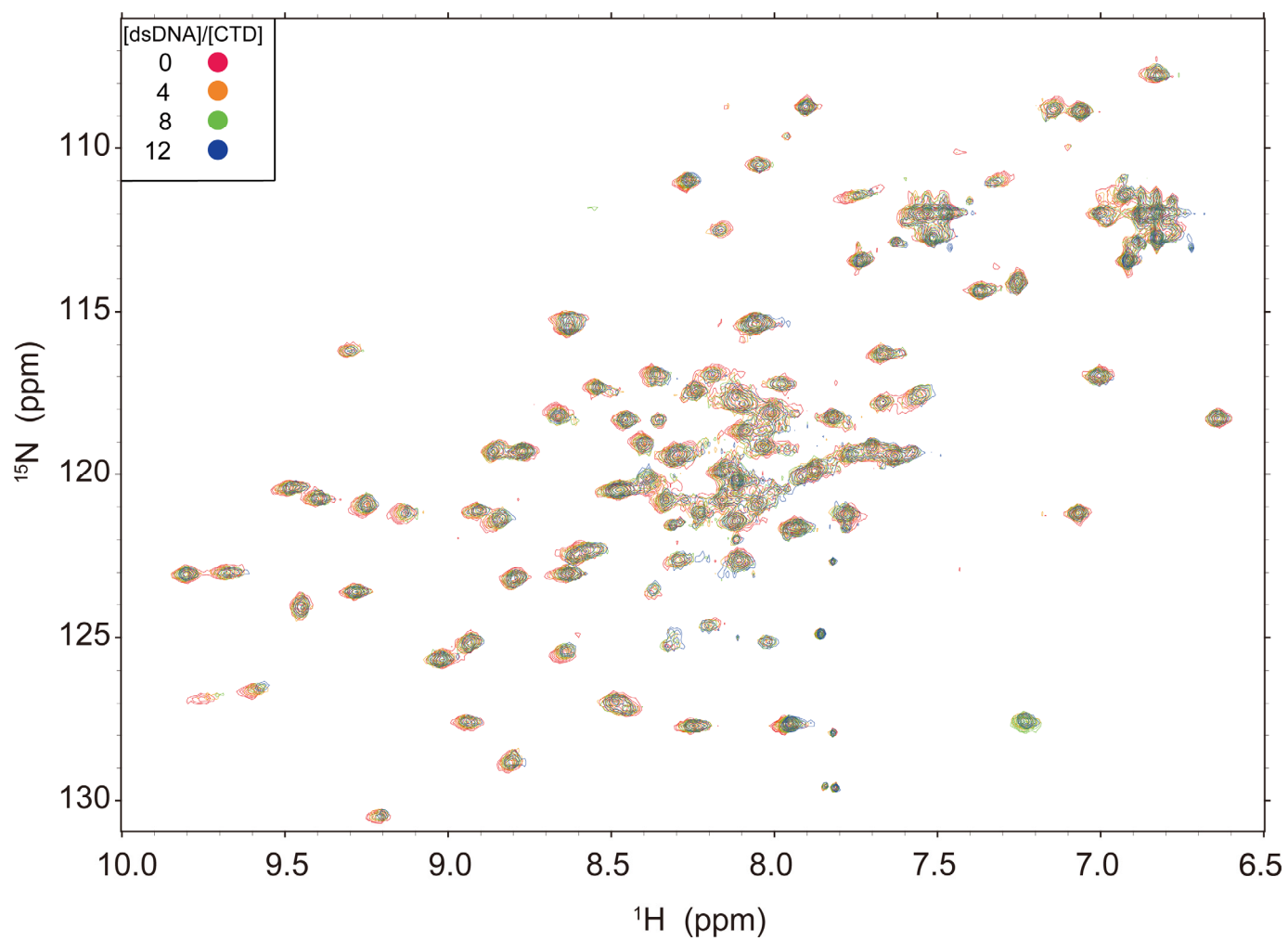

Supplement: Figure S6 — The 1H-15N HSQC spectra of aqMutL-CTD with and without dsDNA. The 1H-15N HSQC spectra of 100 µM aqMutL-CTD at the various dsDNA concentrations were obtained at 313 K in 25 mM Tris-HCl buffer (pH 7.0) containing 25 mM KCl, 5 mM DTT, and 1 mM EDTA. The spectra were overlaid for comparison. (PDF) [file pone.0098554.s006.pdf]

**A**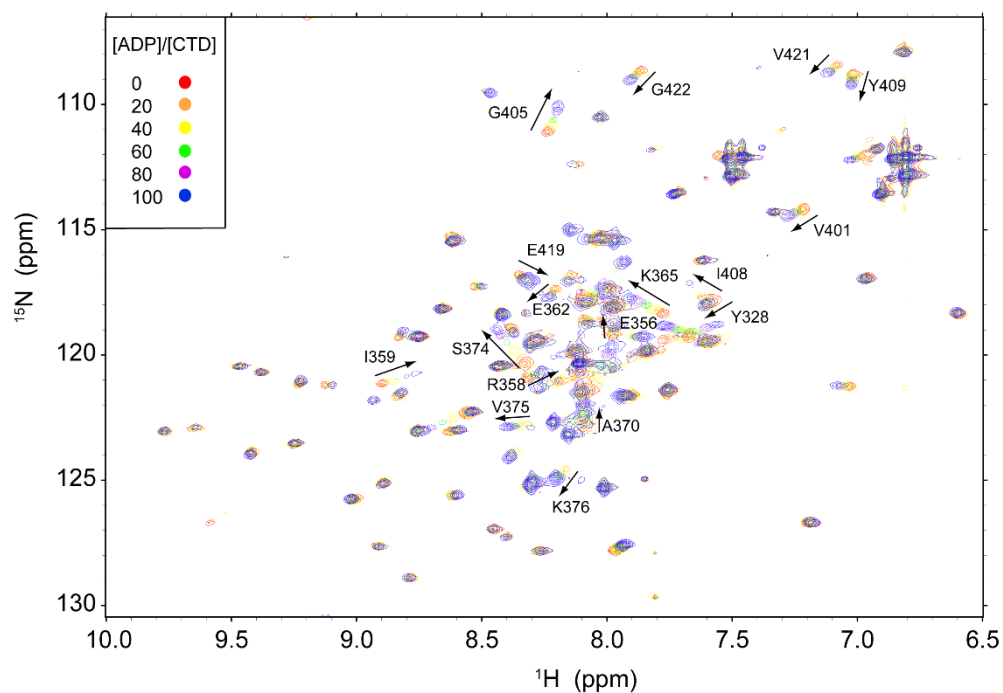**B**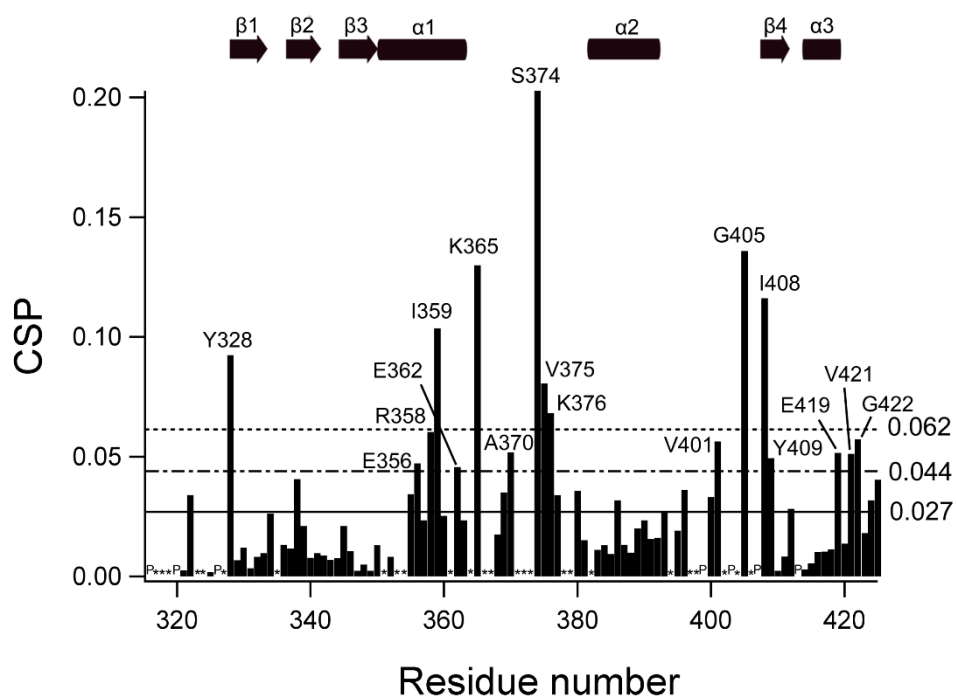**C**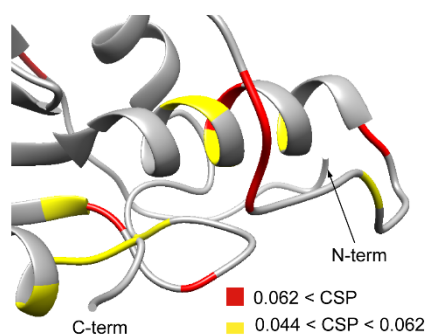

Supplement: Figure S7 — The characterization of interactions between aqMutL-CTD and ADP. (A) The 1H-15N HSQC spectra of 100 µM CTD at various concentrations of ADP are superimposed. The residues displaying significant perturbations are labeled using a one-letter amino acid code and residue numbers. (B) CSPs of the CTD in the presence of 8 mM ADP were plotted against residue numbers. The bottom solid line represents the ″mean value (0.027)″. The middle and upper dotted lines signify ″mean value+0.5× standard deviation (0.044)″ and ″mean value + standard deviation (0.062)″ for significant changes, respectively. (C) The degree of the perturbation was mapped onto the model structure with the color code: Red, CSP>0.062, yellow, 0.062>CSP>0.044. (PDF) [file pone.0098554.s007.pdf]

**A**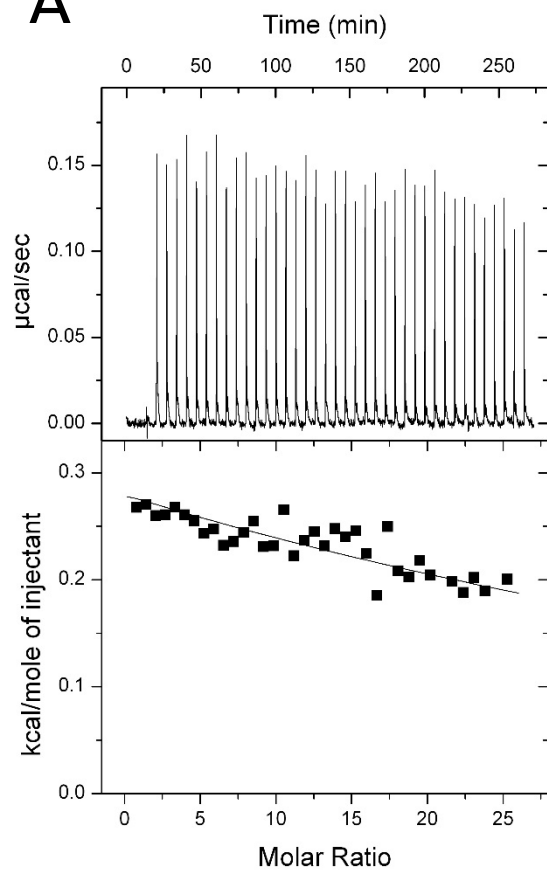**B**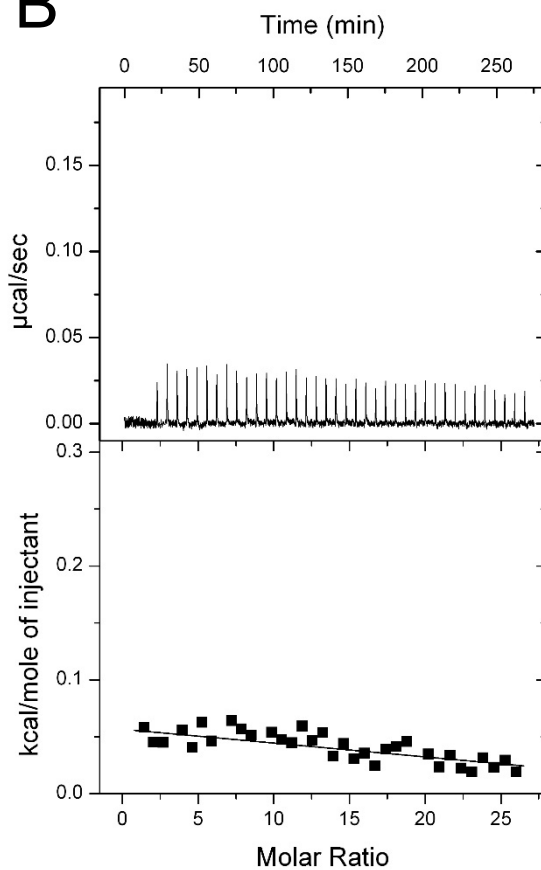

Supplement: Figure S8 — Calorimetric titration of ATP and ADP to aqMutL-CTD. ATP (A) and ADP (B) in the syringe were titrated to CTD in the reaction cell, respectively. Thermograms and binding isotherms are shown in the upper and lower panels, respectively. The corresponding heat of dilution of ATP or ADP titrated to the buffer was used to correct the data. Solid lines to guide the eye in the lower panels are presented. (PDF) [file pone.0098554.s008.pdf]

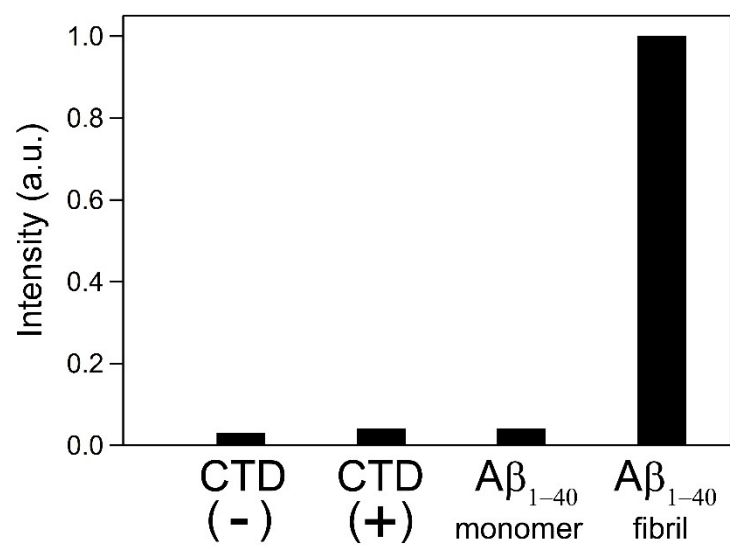

Supplement: Figure S9 — Light scattering measurements of CTD. The normalized intensity of light scattering of CTD solution in the absence (−) and presence (+) of ATP is shown. Light scattering of the monomeric Aβ1–40 peptides and the Aβ1–40 amyloid fibrils of Aβ1–40 peptides which are aggregates is also displayed for comparison. The concentrations of CTD, ATP, and Aβ1–40 peptides were, 16, 160, and 30 µM, respectively. (PDF) [file pone.0098554.s009.pdf]

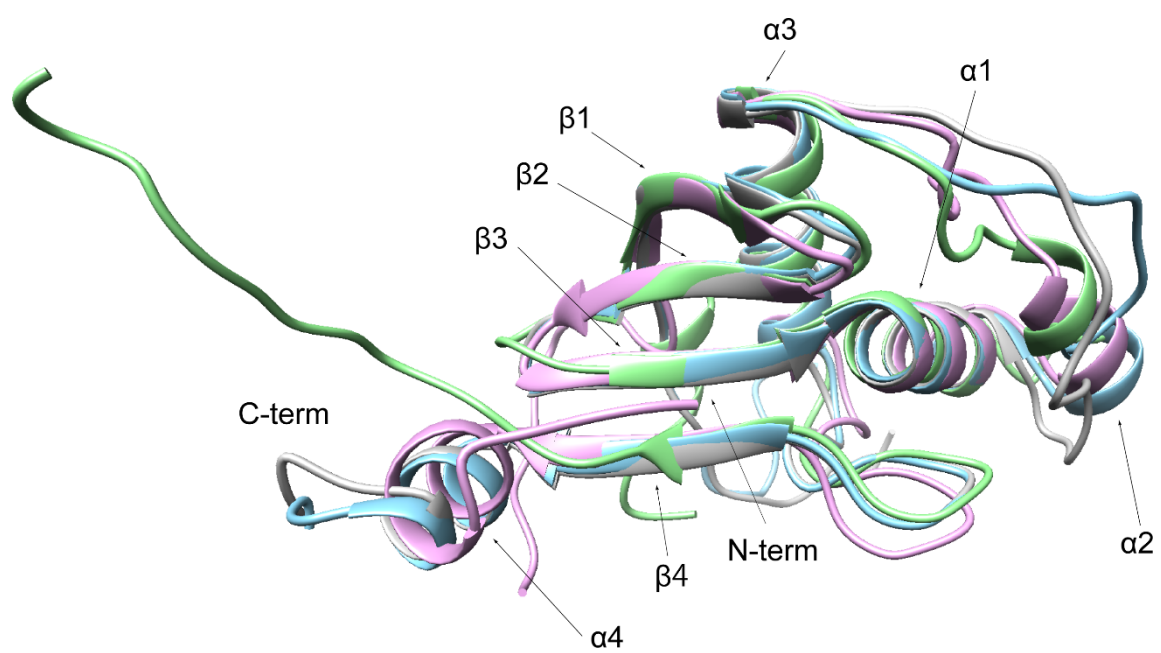

Supplement: Figure S10 — Superimposition of homology-modeled structures of aqMutL-CTD with the various template X-ray structures. The model structure generated by using the template structures of bsMutL-CTD with NMR secondary structure constraints is colored by gray. The model structures templated by using bsMutL-CTD (blue), NgoL MutL-CTD (pink) and PMS1-CTD (green) without NMR constrains are also shown with the monomeric structures. (PDF) [file pone.0098554.s010.pdf]
