# Supplementary material for: NMR Characterization of the Interaction of the Endonuclease Domain of MutL with Divalent Metal Ions and ATP
Source: PLoS One. 2014 Jun 5;9(6):e98554. doi: 10.1371/journal.pone.0098554 (PMC4047009; doi:10.1371/journal.pone.0098554)
Supplement: Figure S5 — Multiple sequence alignments of MutL-CTD. The sequences of the CTDs of MutH-less species (bsMutL, ngMutL, aqMutL, and human MutL homolog PMS2) were displayed using ClustalW (http://clustalw.ddbj.nig.ac.jp). Conserved motifs were highlighted by rectangles. The residue numbers of aqMutL-CTD (K365 and D366) were displayed to note the missing primary sequences of aqMutL-CTD among MutL-homologs. ″*″ indicates perfectly matched residues. (PDF) [file pone.0098554.s005.pdf]

```
CLUSTAL 2.1 multiple sequence alignment


bsMutL_CTD      ---------------------GAMDRVPIMYPIGQMHGTYILAQNENGLYIIDQHAAQER
ngMutL_CTD      TMGSSHHHHHHSSGLVPRGSHSQSELPPLGFAIAQLLGIYILAQAEDSLLLIDMHAAAER
aqMutL_CTD      -----------------PLSQPVKTYKPTYEILGQMDETFILVKDSEYLYFVDQHLLEER
hPMS2_CTD       --------------------------------IGQFNLGFIITKLNEDIFIVDQHATDEK
                                                :.*:   :*:.: .: : ::* *   *:

bsMutL_CTD      IKYEYFR-EKVGEVEPEVQEMIVPLTFHYSTNEALIIEQHKQELESVGVFLESFGSNSYI
ngMutL_CTD      VNYEKMKRQRQENGNLQSQHLLIPVTFAASHEECAALADHAETLAGFGLELSDMGGNTLA
aqMutL_CTD      INYEKLK-----------------------------------------------------
                       365
hPMS2_CTD       YNFEMLQQHTVLQGQRLIAPQTLNLTAVNEAVLIENLEIFRKNGFDFVIDENAPVTERAK
                 ::* ::

bsMutL_CTD      VRCHPAWFPKGEEAELIEEIIQQVLDS-KNIDIKKLREEAAIMMSCKGSIKANRHLRNDE
ngMutL_CTD      VRAAPVMLGKSDVVSLARDVLGELAQVGSSQTIASHENRILATMSCHGSIRAGRRLTLPE
aqMutL_CTD      ----------------------------------------DENLACRISVKAGQKLSEEK
                                                      366
hPMS2_CTD       LISLPTSKNWTFGPQDVDELIFMLSDSPGVMCRPSRVKQMFASRACRKSVMIGTALNTSE
                                                            :*: *:  .  *   :

bsMutL_CTD      IKALLDDLRSTSDPFTCPHGRPIIIHHSTYEMEKMFKRVM
ngMutL_CTD      MNALLRDMENTPRSNQCNHGRPTWVKLTLKELDTLFLRGQ
aqMutL_CTD      IRELIKTWRNLENPHVCPHGRPIYYKIPLREIYEKVGRNY
hPMS2_CTD       MKKLITHMGEMDHPWNCPHGRPTMRHIANLGVISQN----
                :. *:    .   .  * ****   : .   :
```
